# Alpha-protein kinase 3 (*ALPK3*) truncating variants are a cause of autosomal dominant hypertrophic cardiomyopathy

**DOI:** 10.1093/eurheartj/ehab424

**Published:** 2021-07-15

**Authors:** Luis R Lopes, Soledad Garcia-Hernández, Massimiliano Lorenzini, Marta Futema, Olga Chumakova, Dmitry Zateyshchikov, Maria Isidoro-Garcia, Eduardo Villacorta, Luis Escobar-Lopez, Pablo Garcia-Pavia, Raquel Bilbao, David Dobarro, Maria Sandin-Fuentes, Claudio Catalli, Blanca Gener Querol, Ainhoa Mezcua, Jose Garcia Pinilla, Torsten Bloch Rasmussen, Ana Ferreira-Aguar, Pablo Revilla-Martí, Maria Teresa Basurte Elorz, Alicia Bautista Paves, Juan Ramon Gimeno, Ana Virginia Figueroa, Raul Franco-Gutierrez, Maria Eugenia Fuentes-Cañamero, Marina Martinez Moreno, Martin Ortiz-Genga, Jesus Piqueras-Flores, Karina Analia Ramos, Ainars Rudzitis, Luis Ruiz-Guerrero, Ricardo Stein, Mayte Triguero-Bocharán, Luis de la Higuera, Juan Pablo Ochoa, Dad Abu-Bonsrah, Cecilia Y T Kwok, Jacob B Smith, Enzo R Porrello, Mohammed M Akhtar, Joanna Jager, Michael Ashworth, Petros Syrris, David A Elliott, Lorenzo Monserrat, Perry M Elliott

**Affiliations:** Centre for Heart Muscle Disease, Institute of Cardiovascular Science, University College London, 62 Huntley St, London WC1E 6DD, UK; Barts Heart Centre, St. Bartholomew’s Hospital, Barts Health NHS Trust, West Smithfield, London EC1A 7BE, UK; Health in Code S.L., Cardiology and Scientific Department, As Xubias, s/n Edificio O Fortín, 15006 A Coruña, Spain; Centre for Heart Muscle Disease, Institute of Cardiovascular Science, University College London, 62 Huntley St, London WC1E 6DD, UK; Barts Heart Centre, St. Bartholomew’s Hospital, Barts Health NHS Trust, West Smithfield, London EC1A 7BE, UK; Centre for Heart Muscle Disease, Institute of Cardiovascular Science, University College London, 62 Huntley St, London WC1E 6DD, UK; Federal Scientific Clinical Centre of Federal Medical and Biological Agency, 30, Volokolamskoe Shosse, Moscow, Russia; Department of Cardiology, City Clinical Hospital, #17, Volynska st., 7, Moscow, Russia; Federal Scientific Clinical Centre of Federal Medical and Biological Agency, Genetic Laboratory, Moscow, Russia; Inherited Cardiac Disease Unit (CSUR), Biochemistry Department, Instituto de Investigación Biomédica de Salamanca (IBSAL), Complejo Asistencial Universitario de Salamanca, Gerencia Regional de Salud de Castilla y León (SACYL), Medicine Department, Facultad de Medicina, Universidad de Salamanca, Centro de Investigación Biomédica en Red en Enfermedades Cardiovasculares (CIBERCV), Paseo de San Vicente, 58-182, 37007 Salamanca, Madrid, Spain; Inherited Cardiac Disease Unit (CSUR), Cardiology Department, Instituto de Investigación Biomédica de Salamanca (IBSAL), Complejo Asistencial Universitario de Salamanca, Gerencia Regional de Salud de Castilla y León (SACYL), Medicine Department, Facultad de Medicina, Universidad de Salamanca, Centro de Investigación Biomédica en Red en Enfermedades Cardiovasculares (CIBERCV), Paseo de San Vicente, 58-182, 37007 Salamanca and Av. Monforte de Lemos, 3-5. Pabellón 11. Planta 0 28029, Madrid, Spain; Heart Failure and Inherited Cardiac Diseases Unit, Department of Cardiology, Hospital Universitario Puerta de Hierro, CIBERCV, Av. Monforte de Lemos, 3-5. Pabellón 11. Planta 0 28029 and Calle Joaquín Rodrigo, 1, 28222 Majadahonda, Madrid, Spain; European Reference Network for Rare and Low Prevalence Complex Diseases of the Heart (ERN-GUARDHEART); Heart Failure and Inherited Cardiac Diseases Unit, Department of Cardiology, Hospital Universitario Puerta de Hierro, CIBERCV, Av. Monforte de Lemos, 3-5. Pabellón 11. Planta 0 28029 and Calle Joaquín Rodrigo, 1, 28222 Majadahonda, Madrid, Spain; European Reference Network for Rare and Low Prevalence Complex Diseases of the Heart (ERN-GUARDHEART); Universidad Francisco de Vitoria (UFV), Pozuelo de Alarcón, Carretera Pozuelo a Majadahonda, Km 1.800, 28223 Madrid, Spain; Heart Failure and Pulmonary Hypertension Unit, Hospital Alvaro Cunqueiro, Complexo Hospitalario Universitario de Vigo, Estrada de Clara Campoamor, 341, 36213 Vigo, Pontevedra, Spain; Heart Failure and Pulmonary Hypertension Unit, Hospital Alvaro Cunqueiro, Complexo Hospitalario Universitario de Vigo, Estrada de Clara Campoamor, 341, 36213 Vigo, Pontevedra, Spain; Hospital Clínico Universitario de Valladolid, Cardiology, Av. Ramón y Cajal, 3, 47003 Valladolid, Spain; Osakidetza Basque Health Service, Cruces University Hospital, Department of Genetics, Biocruces Bizkaia Health Research Institute, Cruces Plaza, 48903 Barakaldo, Bizkaia, Spain; Osakidetza Basque Health Service, Cruces University Hospital, Department of Genetics, Biocruces Bizkaia Health Research Institute, Cruces Plaza, 48903 Barakaldo, Bizkaia, Spain; Heart Failure and Familial Heart Diseases Unit, Cardiology Service, Hospital Universitario Virgen de la Victoria, IBIMA, Campus de Teatinos, S/N, 29010 Málaga, Spain; Centro de Investigación Biomédica en Red de Enfermedades Cardiovasculares (CIBERCV), Av. Monforte de Lemos, 3-5. Pabellón 11. Planta 0 28029 Madrid, Spain; Heart Failure and Familial Heart Diseases Unit, Cardiology Service, Hospital Universitario Virgen de la Victoria, IBIMA, Campus de Teatinos, S/N, 29010 Málaga, Spain; Centro de Investigación Biomédica en Red de Enfermedades Cardiovasculares (CIBERCV), Av. Monforte de Lemos, 3-5. Pabellón 11. Planta 0 28029 Madrid, Spain; Department of Cardiology, Aarhus University Hospital, Palle Juul-Jensens Boulevard 99 DK-8200 Aarhus, Denmark; Inherited Cardiac Diseases Unit, Cardiology Department, Hospital Clínico Universitario Lozano Blesa, Avda, Calle de San Juan Bosco, 15, 50009 Zaragoza, Spain; Inherited Cardiac Diseases Unit, Cardiology Department, Hospital Clínico Universitario Lozano Blesa, Avda, Calle de San Juan Bosco, 15, 50009 Zaragoza, Spain; Complejo Hospitalario de Navarra, Calle de Irunlarrea, 3, 31008 Pamplona, Navarra, Spain; Hospital Universitario San Cecilio Granada, Av. del Conocimiento, s/n, 18016 Granada, Cardiology; European Reference Network for Rare and Low Prevalence Complex Diseases of the Heart (ERN-GUARDHEART); Hospital Clínico Universitario Virgen de la Arrixaca, Inherited Cardiac Diseases Unit, Department of Cardiology, Ctra. Madrid-Cartagena, s/n, 30120 El Palmar, Murcia, Spain; Hospital Privado Universitario de Córdoba, Naciones Unidas 346, Córdoba, Argentina; Cardiology Department, Hospital Universitario Lucus Augusti, Lugo Biodiscovery HULA-USC Research Group, Institute for Health Research of Santiago de Compostela IDIS, s/n A, Travesía da Choupana, 15706 Santiago de Compostela, A Coruña; Hospital Universitario Infanta Cristina, Cardiology, Av. de Elvas, s/n, 06080 Badajoz, Spain; Hospital General Elche, Carrer Almazara, 11, 03203 Elche, Alicante; Health in Code S.L., Scientific Department, As Xubias, s/n Edificio O Fortín, 15006 A Coruña, Spain; Cardiology Department, Inherited Cardiovascular Diseases Unit, Hospital General Universitario de Ciudad Real, Calle Obispo Rafael Torija, s/n, 13005 Ciudad Real, Spain; Hospital Centenario, Urquiza 3101, S2002 KDT, Santa Fe, Rosario, Argentina; Pauls Stradins Clinical University Hospital, Pilsoņu iela 13, Zemgales priekšpilsēta, Rīga, LV-1002, Latvia; Hospital Universitario Marqués de Valdecilla (IDIVAL), Av. de Valdecilla, 25, 39008 Santander, Spain; School of Medicine, Universidade Federal do Rio Grande do Sul, Porto Alegre, Av. Paulo Gama, 110 Secretaria de Comunicação Social – 8º andar – Reitoria – Farroupilha, Porto Alegre – RS 90040-060, Brazil; Cardiology Department, Inherited Cardiovascular Diseases Unit, Hospital General Universitario de Ciudad Real, Calle Obispo Rafael Torija, s/n, 13005 Ciudad Real, Spain; Health in Code S.L., Scientific Department, As Xubias, s/n Edificio O Fortín, 15006 A Coruña, Spain; Heart Failure and Inherited Cardiac Diseases Unit, Department of Cardiology, Hospital Universitario Puerta de Hierro, CIBERCV, Av. Monforte de Lemos, 3-5. Pabellón 11. Planta 0 28029 and Calle Joaquín Rodrigo, 1, 28222 Majadahonda, Madrid, Spain; European Reference Network for Rare and Low Prevalence Complex Diseases of the Heart (ERN-GUARDHEART); Murdoch Research Childrens Research Institute, Royal Melbourne Hospital, Parkville, VIC 3052, Australia; Murdoch Research Childrens Research Institute, Royal Melbourne Hospital, Parkville, VIC 3052, Australia; Murdoch Research Childrens Research Institute, Royal Melbourne Hospital, Parkville, VIC 3052, Australia; Murdoch Research Childrens Research Institute, Royal Melbourne Hospital, Parkville, VIC 3052, Australia; Dept. of Physiology, University of Melbourne, Parkville, VIC 3052, Australia; Centre for Heart Muscle Disease, Institute of Cardiovascular Science, University College London, 62 Huntley St, London WC1E 6DD, UK; Barts Heart Centre, St. Bartholomew’s Hospital, Barts Health NHS Trust, West Smithfield, London EC1A 7BE, UK; Centre for Heart Muscle Disease, Institute of Cardiovascular Science, University College London, 62 Huntley St, London WC1E 6DD, UK; Department of Histopathology, Great Ormond St Hospital for Children, London WC1N 3NN, UK; Centre for Heart Muscle Disease, Institute of Cardiovascular Science, University College London, 62 Huntley St, London WC1E 6DD, UK; Murdoch Research Childrens Research Institute, Royal Melbourne Hospital, Parkville, VIC 3052, Australia; Dept. of Physiology, University of Melbourne, Parkville, VIC 3052, Australia; Health in Code S.L., Scientific Department, As Xubias, s/n Edificio O Fortín, 15006 A Coruña, Spain; Centre for Heart Muscle Disease, Institute of Cardiovascular Science, University College London, 62 Huntley St, London WC1E 6DD, UK; Barts Heart Centre, St. Bartholomew’s Hospital, Barts Health NHS Trust, West Smithfield, London EC1A 7BE, UK

**Keywords:** *ALPK3*, Hypertrophic cardiomyopathy, Genetics

## Abstract

**Aims:**

The aim of this study was to determine the frequency of heterozygous truncating *ALPK3* variants (*ALPK3*tv) in patients with hypertrophic cardiomyopathy (HCM) and confirm their pathogenicity using burden testing in independent cohorts and family co-segregation studies.

**Methods and results:**

In a discovery cohort of 770 index patients with HCM, 12 (1.56%) were heterozygous for *ALPK3*tv [odds ratio(OR) 16.11, 95% confidence interval (CI) 7.94–30.02, *P* = 8.05e−11] compared to the Genome Aggregation Database (gnomAD) population. In a validation cohort of 2047 HCM probands, 32 (1.56%) carried heterozygous *ALPK3*tv (OR 16.17, 95% CI 10.31–24.87, *P* < 2.2e−16, compared to gnomAD). Combined logarithm of odds score in seven families with *ALPK3*tv was 2.99. In comparison with a cohort of genotyped patients with HCM (*n* = 1679) with and without pathogenic sarcomere gene variants (SP+ and SP−), *ALPK3*tv carriers had a higher prevalence of apical/concentric patterns of hypertrophy (60%, *P* < 0.001) and of a short PR interval (10%, *P* = 0.009). Age at diagnosis and maximum left ventricular wall thickness were similar to SP− and left ventricular systolic impairment (6%) and non-sustained ventricular tachycardia (31%) at baseline similar to SP+. After 5.3 ± 5.7 years, 4 (9%) patients with *ALPK3*tv died of heart failure or had cardiac transplantation (log-rank *P* = 0.012 vs. SP− and *P* = 0.425 vs. SP+). Imaging and histopathology showed extensive myocardial fibrosis and myocyte vacuolation.

**Conclusions:**

Heterozygous *ALPK3*tv are pathogenic and segregate with a characteristic HCM phenotype.

## Graphical Abstract

**Figure:**
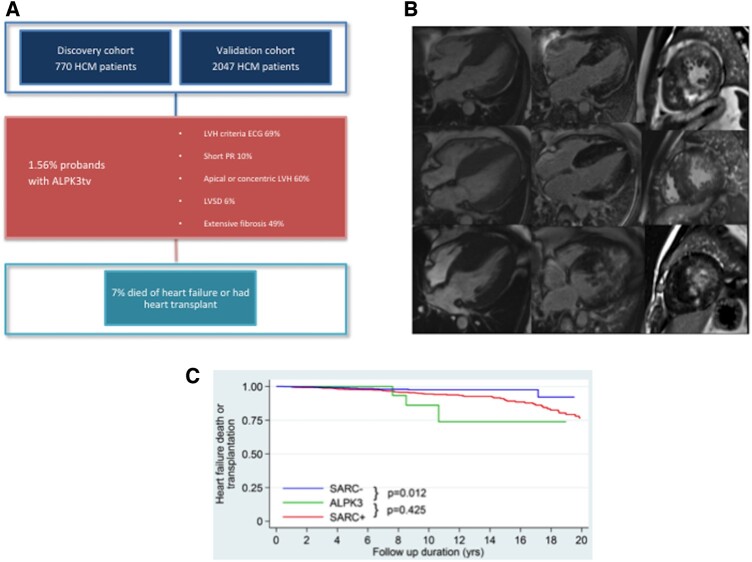
Truncating variants in *ALPK3* are a cause of 1–2% of autosomal dominant hypertrophic cardiomyopathy and are associated with a phenotype characterized by extensive fibrosis and a predominantly concentric or apical pattern of left ventricular hypertrophy without left ventricular outflow tract obstruction. (*A*) Main phenotype characteristics and outcomes. *ALPK3*tv, alpha-protein kinase 3-truncating variants; CK, creatine kinase; HCM, hypertrophic cardiomyopathy; ICD, implantable cardioverter–defibrillator; LVH, left ventricular hypertrophy; LVSD, left ventricular systolic dysfunction; SCD, sudden cardiac death. (*B*) Cardiac magnetic resonance imaging showing the prevalent phenotype of severe mid to apical hypertrophy and extensive late gadolinium enhancement in three patients. Left to right: 4-chamber view end-diastole cine image, 4-chamber view late gadolinium enhancement image, short axis late gadolinium enhancement image. (*C*) Kaplan–Meier analysis comparing incidence of an outcome of heart failure death and transplant between *ALP3*tv patients, sarcomere positive and sarcomere negative. SARC+: sarcomere positive; SARC−: sarcomere negative.

**See page 3074 for the editorial comment on this article (doi:10.1093/eurheartj/ehab415)**

## Introduction

Hypertrophic cardiomyopathy (HCM)—defined as left ventricular hypertrophy (LVH) unexplained by abnormal loading conditions—is a myocardial disease affecting 1 in 500 of the general population and is a major cause of sudden cardiac death (SCD), heart failure, and stroke.^1^ HCM is most frequently inherited as an autosomal dominant genetic trait caused by pathogenic variants in cardiac sarcomere genes, but the yield of clinical genetic testing is no more than 60%, even in patients with a family history of the disease.^2^ This gap in knowledge exposes individuals and families to uncertainty about their future health and hampers efforts to develop novel disease modifying therapies.

Alpha-protein kinase 3 (*ALPK3*), located on chromosome 15q25.2, has recently emerged as a possible candidate gene in cardiomyopathy.^3^ Bi-allelic truncating variants in *ALPK3* (*ALPK3*tv) have been reported in small paediatric case series, presenting with a complex phenotype of dilated cardiomyopathy (DCM) often evolving into HCM with poor systolic function^3–8^ and common variants in *ALPK3* have been associated with DCM and more recently HCM in genome-wide approaches.^9–13^ A case report has described a family with HCM caused by a heterozygous *ALPK3* variant^14^ and *ALPK3*tv were found to be enriched in a mixed cohort of patients with HCM and DCM.^8^

In this study, we sought to determine the frequency of heterozygous rare *ALPK3*tv in a discovery cohort of patients with HCM investigated with whole-exome sequencing and to establish their pathogenicity by means of burden testing in independent cohorts and family co-segregation studies. The findings show that heterozygous *ALPK3tv* cause a severe clinical phenotype in adults with HCM.

## Methods

### Discovery cohort—study population and genetic analyses

The discovery cohort comprised 770 consecutively evaluated unrelated patients with HCM referred to the Inherited Cardiovascular Disease unit at St. Bartholomew’s Hospital, London, UK, and before 2015, to the Inherited Cardiovascular Disease Unit at The Heart Hospital, UCLH, London, UK. The samples used in this study were collected from 2013 to 2018. All patients gave written informed consent, and the study was approved by the regional ethics committee (15/LO/0549). Clinical evaluation was as previously described.^15^ ^,^ ^16^ HCM was diagnosed according to current European Society of Cardiology guidelines.^1^ Patients with previously confirmed HCM phenocopies were excluded from the study.

DNA extraction, library preparation, whole-exome sequencing, variant calling, and annotation were performed as described previously^16^; variants identified with a minor allele frequency more than or equal to 0.0001 in the Genome Aggregation Database (gnomAD)^17^ (in any population) were removed from further analysis. The analysis of large rearrangements was performed using a read-depth strategy (ExomeDepth).^18^ Prioritized variants were confirmed by conventional automated (Sanger) DNA sequencing.^16^

### Validation in a multicentre cohort using enrichment analysis and familial co-segregation

From 2018 to 2020, *ALPK3* was sequenced using next-generation sequencing in 4904 consecutive unrelated probands with inherited cardiac conditions referred for molecular genetic diagnosis at Health in Code. This cohort encompassed different cardiovascular phenotypes established by each referring centre: HCM (*n* = 2047), DCM (*n* = 746), arrhythmogenic cardiomyopathy (ACM, *n* = 435), non-compaction cardiomyopathy (*n* = 313), restrictive cardiomyopathy (*n* = 41), and undefined cardiomyopathy (*n* = 121). An additional 1059 index cases with no evidence of structural cardiac disease (channelopathies and aortic diseases) were used as controls. The remaining 142 individuals in this cohort had an unestablished phenotype.

Patients were referred mainly from centres in Spain, the UK, Denmark, Russia, Latvia, Brazil, and Argentina. Ethical approval and patient consent was obtained from participating centres.

Sequencing, variant filtering, and classification were conducted as previously described.^19^ Coding exons and intronic boundaries of 261 genes related to inherited cardiovascular diseases and SCD (see Supplementary material online, *Table S1*) were captured using a custom probe library (SureSelect Target Enrichment Kit for Illumina paired-end multiplexed sequencing method, Agilent Technologies, Santa Clara, CA, USA) and sequenced using the HiSeq 1500 platform (Illumina, San Diego, CA, USA) following lllumina protocols. The read depth of every nucleotide of genes related to the referring phenotype (including *ALPK3*) was >30 fold (mean 250× to 400×). Exons that did not fulfil this standard were additionally sequenced using the Sanger method. Predicted loss-of-function variants (frameshift, nonsense, and consensus splice site variants) in the latest reported *ALPK3* isoform (NM_020778.5; NP_065829.4) were selected for further analysis. Bioinformatics analysis was performed by means of a custom pipeline including software for variant calling, genotyping, and annotation.

The statistical significance of the *ALPK3* variant enrichment in cases vs. controls was tested using Fisher’s exact statistics. The enrichment analysis was performed in the entire validation cohort (regardless of whether the patients were finally recruited or not) to avoid inclusion bias.

To check for technical artefacts in the enrichment analysis, we have evaluated their comparability through the quantification of the burden of rare synonymous variants in *ALPK3*tv between every compared group and no statistical differences were observed (results presented in the Supplementary material online).

HCM probands carrying candidate variants in *ALPK3* and none in other genes were invited to participate in segregation studies. Clinical and genetic familial cascade screening was performed following written informed consent in those who agreed to participate. All the participating families belonged to the validation cohort.

**Figure 1:**
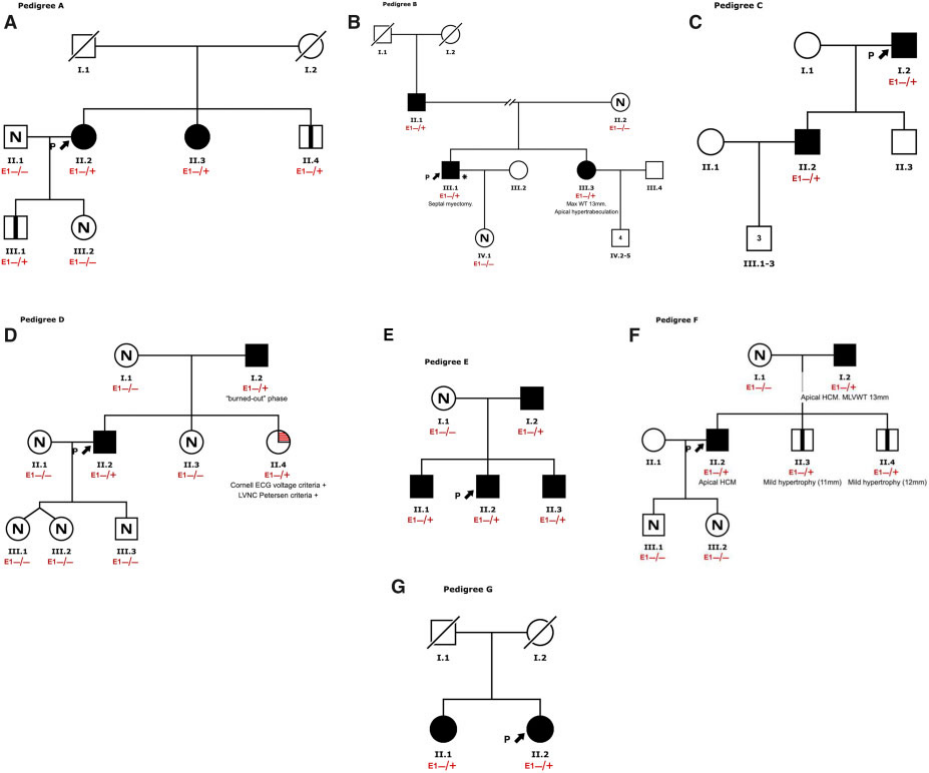
Pedigrees for the families in which co-segregation analyses were performed. (*A*) Proband #36 (p.Glu1146Glyfs*12); (*B*) proband #23 (p.Trp1563*); (*C*) proband #37 (p.Glu1179Argfs*93); (*D*) proband #18 (p.Pro45Alafs*37); (*E*) proband #32 (p.Pro45Alafs*37); (*F*) proband #43 (p.Glu1098*); and (*G*) proband #38 (p.Lys184*). Arrows indicate the probands. Filled symbols, affected. N: not affected. Squares: males. Circles: females. Vertical bar inside symbol: ALPK3tv carrier. WT,: wall thickness; LVNC,: left ventricular non-compaction.

We calculated two-point logarithm of the odds (LOD) scores for seven informative families with HCM (*Figure 1* and Supplementary material online, *Table S2*) by using the PARAMLINK package for R software, computed with the settings θ = 0, phenocopy rate = 0.002 (prevalence of the disease in the general population) and indicated disease penetrance (0.80 and 0.95).^20^ An indeterminate status was assigned to family members ≤45 years of age who did not meet clinical criteria for HCM and to family members with confounding cardiac diagnoses.

**Figure 2:**
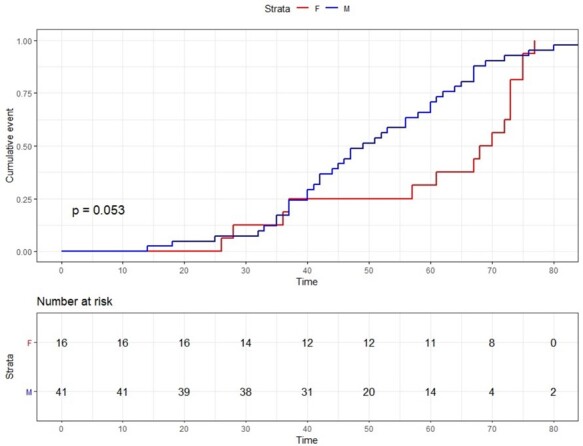
Kaplan–Meier estimates for age at diagnosis of HCM in probands and relatives with alpha-protein kinase 3-truncating variants (*ALPK3*tv). The analysis included non-affected relatives. “Time” represents age in years.

**Figure 3:**
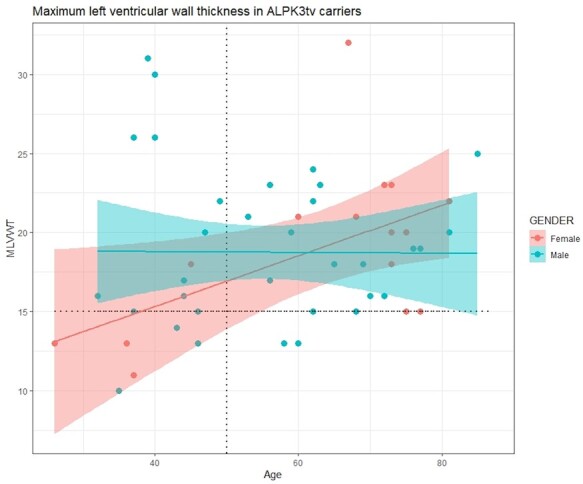
Scatter plot of age vs. maximum left ventricular wall thickness in *ALPK3*tv carriers. This graph was based on information available on all heterozygous carriers from both cohorts (validation and discovery, probands and relatives). The maximum left ventricular wall thickness was taken from magnetic resonance (or echocardiography results when magnetic resonance study was not performed). A positive correlation between maximum left ventricular wall thickness and age is seen for females. MLVWT, maximum left ventricular wall thickness.

Kaplan–Meier curves for age at diagnosis were depicted with the information on both cohorts (validation and discovery) and were computed by means of the ‘survfit’ function in R. In *Figures 2 and 3*, we represent all individuals harbouring ALPK3tv variants (both probands and relatives).

### Clinical characteristics and outcomes of patients with ALPK3tv in comparison to genotyped patients with HCM

Patients with *ALPK3tv* (probands and affected relatives from discovery and validation cohorts) with available follow-up data were compared to adult patients (≥18 years of age) with HCM with an available genotype and follow-up data, evaluated between 1986 and 2019.

Variants were classified as pathogenic, likely pathogenic, unknown significance, or likely benign/benign using the current criteria of the American College of Medical Genetics and Genomics.^21^ Patients with ≥1 pathogenic or likely pathogenic variant were designated as sarcomere positive, while those with no pathogenic or likely pathogenic variants were designated as sarcomere negative.

A detailed description of this cohort and definitions of the phenotype parameters analysed are provided in the Supplementary material online, *Methods*.

Means for age at diagnosis, maximal wall thickness, and left atrial diameter were compared with one-way ANOVA and Tukey HSD or Games–Howell *post hoc* analysis, as appropriate. The frequency of categorical variables (male sex, ECG parameters, left ventricular systolic dysfunction defined as left ventricular ejection fraction <50% and non-sustained ventricular tachycardia) was compared by Chi-square or Fisher’s exact test, as appropriate. Survival analysis for heart failure death and transplant was carried out using the Kaplan–Meier method and groups were compared using the log-rank test.

### Histopathology analysis

Cardiac tissue was available and re-analysed from two patients who had a septal myectomy. Tissue was formalin-fixed and processed to paraffin wax. Sections were cut at 4 μm and stained with haematoxylin and eosin, Masson trichrome, and periodic acid-Schiff (PAS) stains. Immunohistochemistry was performed for desmin and plakoglobin. We additionally report the clinical findings for a skeletal muscle biopsy (left quadriceps) and cardiac muscle biopsy (myectomy) for two other patients (Supplementary material online, *Table S3*), for whom tissue was not available to re-analyse.

## Results

The discovery cohort comprised 770 patients with HCM, aged 49.3 ± 15.9 years at diagnosis (median 51.6); 515 (67%) were male; 378 out of 589 where ethnicity was stated (64%) were white; 246 (32%) had rare variants in eight sarcomeric genes robustly associated with HCM (*MYH7, MYBPC3, TNNT2, TNNI3, MYL2, MYL3, TPM1, ACTC1*). Twenty-one patients carried multiple rare sarcomere variants.

### ALPK3-truncating variants

**Figure 4:**
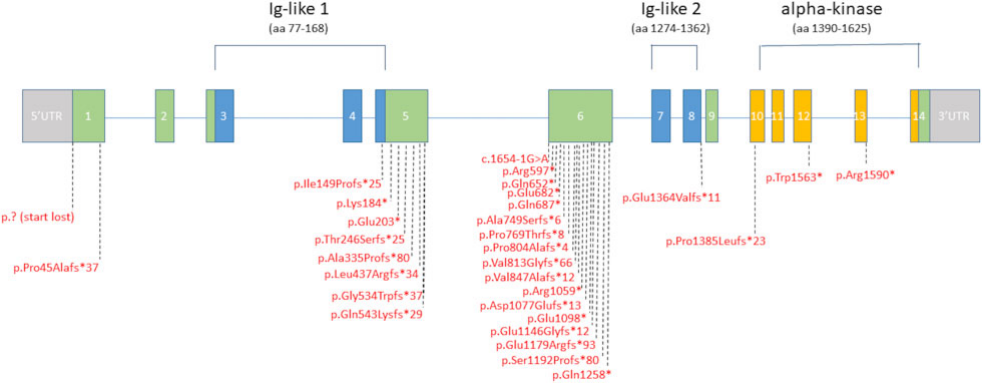
Distribution of rare *ALPK3*-truncating variants along the gene.

Supplementary material online, *Table S3* summarizes the genetic characteristics of patients with *ALPK3*tv. *Figure 4* represents the distribution of *ALPK3*tv along the gene.

#### Discovery cohort

In the discovery cohort, 12 patients (1.56%) were heterozygous carriers of an *ALPK3*tv. All these variants were confirmed with Sanger sequencing. These variants were enriched when compared to the prevalence of truncating variants in the gnomAD population (12/770 HCM-discovery cohort vs. 74/75431 gnomAD) with an odds ratio (OR) of 16.11 [95% confidence interval (CI): 7.94–30.02; *P* 8.05e−11]. No copy-number variants in *ALPK3* were detected. Three patients were found to harbour variants of uncertain significance (VUS) in sarcomere genes (Supplementary material online, *Table S3*): one patient with an *MYH7* missense variant (#10) and two patients with an *MYBPC3* missense variant (#1; #11). No proband was found to carry a second rare missense *ALPK3* variant.

#### Validation cohort

In the validation cohort, a total of 24 *ALPK3*tv were identified in 36 probands in simple heterozygosity, out of 4904 (0.86%) consecutive unrelated index cases who were sequenced with a genetic library including this gene. HCM was the diagnosis in 32 out of the 36 probands. The remaining 4 cases had other phenotypes: DCM (*n* = 1), ACM (*n* = 1), restrictive cardiomyopathy (*n* = 1) and Brugada syndrome (*n* = 1). From these 36 identified patients, 30 were finally recruited to this study, diagnosed with HCM (*n* = 29) and ACM (*n* = 1). One patient was homozygous for the truncating *ALPK3* variant p.Trp1563* (c.4689delG); this case was not included in the enrichment analysis (where only heterozygous variants were analysed).

The prevalence of *ALPK3*tv found in simple heterozygosity was significantly higher in the HCM cohort (32/2047; 1.56%) than in disease control subjects/non-cardiomyopathy controls (1/1059; 0.09%), non-HCM cases (4/2857; 0.14%) and gnomAD database (74/75431; 0.1%), with an OR of 16.8 (95% CI: 2.79–682.77; *P* = 2.375e−05), 11.32 (95% CI: 4.01–44.09; *P* = 8.01e−09), and 16.17 (95% CI: 10.31–24.87; *P* = 2.2e−16), respectively.

Two of the HCM patients with *ALPK3*tv in simple heterozygosity in the validation cohort were found to carry a second variant in a sarcomeric gene in digenic heterozygosis (Supplementary material online, *Table S3*) [*MYBPC3* VUS (p.Pro677Ser) (# 17), *MYH7* pathogenic variant (p.Arg869Cys) (# 39)]. One case diagnosed with restrictive physiology was also a carrier of a *FLNC* variant (p.Gly2011Arg) (# 30). The remaining cases had no other candidate variant as a potential cause for their phenotype.

#### Recurrent variants

Four variants were particularly frequent in the discovery and validation cohorts combined: p.Arg1059* (five probands from different geographic origins, including the UK, Spain, and Latvia), p.Glu1179Argfs*93 (three probands, all from Spain), p.Trp1563* (c.4689delG, three probands, from Spain, including one patient homozygous for this variant).

Three (# 8, # 13, # 14) of the five patients harbouring the variant p.Arg1059* also had a missense variant, p.Arg86Trp. This fact, along with their similar frequencies in the gnomAD population, suggests that these two variants are in linkage disequilibrium and part of a haplotype; the effect of the nonsense variant usually prevails in these cases.

### Clinical characterization of HCM patients with heterozygous ALPK3tv and comparison with a genotyped HCM cohort

*Table 1* and Supplementary material online, *Tables S3–S5* summarize the clinical characteristics of the 51 patients (probands and relatives) with *ALPKtv* from both discovery and validation cohorts. In *Table 1*, the phenotype and outcomes are compared with sarcomere positive and sarcomere negative patients.

**Table 1:** Comparison of the main baseline and heart failure outcomes between genotype subgroups

	Sarcomere −	Sarcomere +	*ALPK3tv*	*P*-value	*P*-value	
	(*N* = 794)	(*N* = 885)	(*N* = 51)		Sarc− vs. *ALPK*3	Sarc+ vs. *ALPK*3
Age at diagnosis (years)	54 ± 13.8	41.1 ± 14.5	56 ± 15.9	<0.001	0.635	<0.001
Male sex	577 (72.7%)	527 (59.5%)	35 (68.6%)	<0.001	0.531	0.198
ECG						
LVH	132/187 (70.6%)	177/334 (53%)	34/49 (69.4%)	<0.001	0.870	0.031
Short PR	8/551 (1.5%)	14/633 (2.2%)	4/41 (9.8%)	0.009	0.007	0.019
IVCD	66/318 (20.8%)	129/431 (29.9%)	1/49 (2%)	<0.001	0.002	0.011
RBBB	34/318 (10.7%)	34/431 (7.9%)	6/49 (12.2%)		0.745	0.279
LBBB	46/318 (14.5%)	25/431 (5.8%)	3/49 (6.1%)		0.110	1
Echocardiogram						
Max wall thickness (mm)^a^	18 ± 4	19 ± 5	18 ± 5	<0.001	0.993	0.451
LVH morphology						
Asymmetric	517 (65.1%)	768 (86.8%)	20 (40%)	<0.001	<0.001	<0.001
Concentric	104 (13.1%)	73 (8.2%)	15 (30%)			
Apical	173 (21.8%)	44 (5%)	15 (30%)			
LVEF	66 ± 9	66 ± 10	67 ± 10	0.409	0.824	0.921
LVEF <50%	28 (3.5%)	38 (4.3%)	3/49 (6.1%)	0.462	0.418	0.470
Left atrium (mm)	43 ± 7	43 ± 8	44 ± 7	0.748	0.735	0.784
Non-sustained VT (baseline)	139/669 (20.8%)	198/809 (24.5%)	11/35 (31.4%)	0.119	0.134	0.351
Follow-up duration (years)	6.9 ± 4.5	9.1 ± 6.4	5.3 ± 5.7 (*N* = 45)	<0.001	0.153	<0.001
Heart failure death	12 (1.5%)	33 (3.7%)	2 (4.4%)			
Transplant	1 (0.1%)	28 (3.2%)	1 (2.2%)			

IVCD, non-specific intraventricular conduction delay; LBBB, left bundle branch block; LVEF, left ventricular ejection fraction; LVH, left ventricular hypertrophy; RBBB, right bundle branch block; VT, ventricular tachycardia.

a CMR when echo not diagnostic. ALPK3tv includes probands and affected relatives. Means compared with one-way ANOVA. Frequencies compared by chi-square or Fisher’s exact test, as appropriate.

#### Demographic, ECG, and imaging characteristics

The age at diagnosis (56 ± 15.9 years) and sex distribution (69% males) of HCM patients with *ALPK3*tv were similar to sarcomere-negative and higher than sarcomere-positive patients.

From the initial WES discovery cohort, 6 out of 12 patients were white and 5 South Asian. We have stratified the enrichment analysis in the discovery cohort by ethnicity as follows: for European (non-Finnish), 6/378 (1.59%) compared to 49/33718 (0.15%), OR 10.92, 95% CI: 3.79–25.72, *P* = 3.54e−05; for South Asian, 5/88 (5.68%) compared to 3/2380 (0.13%), OR 44.75, 95% CI: 8.56–291.59, *P* = 3.44e−06. In the validation cohort, one proband was black and the remainder were white.

The prevalence of voltage criteria for LVH (69%) in carriers of *ALPK3*tv was higher compared to sarcomere-positive patients and similar to sarcomere-negative patients. Short PR was more prevalent (10%) than for any of the other genotyped subgroups, as was the proportion of an apical/concentric hypertrophy pattern (60%) compared to asymmetric septal hypertrophy.

**Figure 5:**
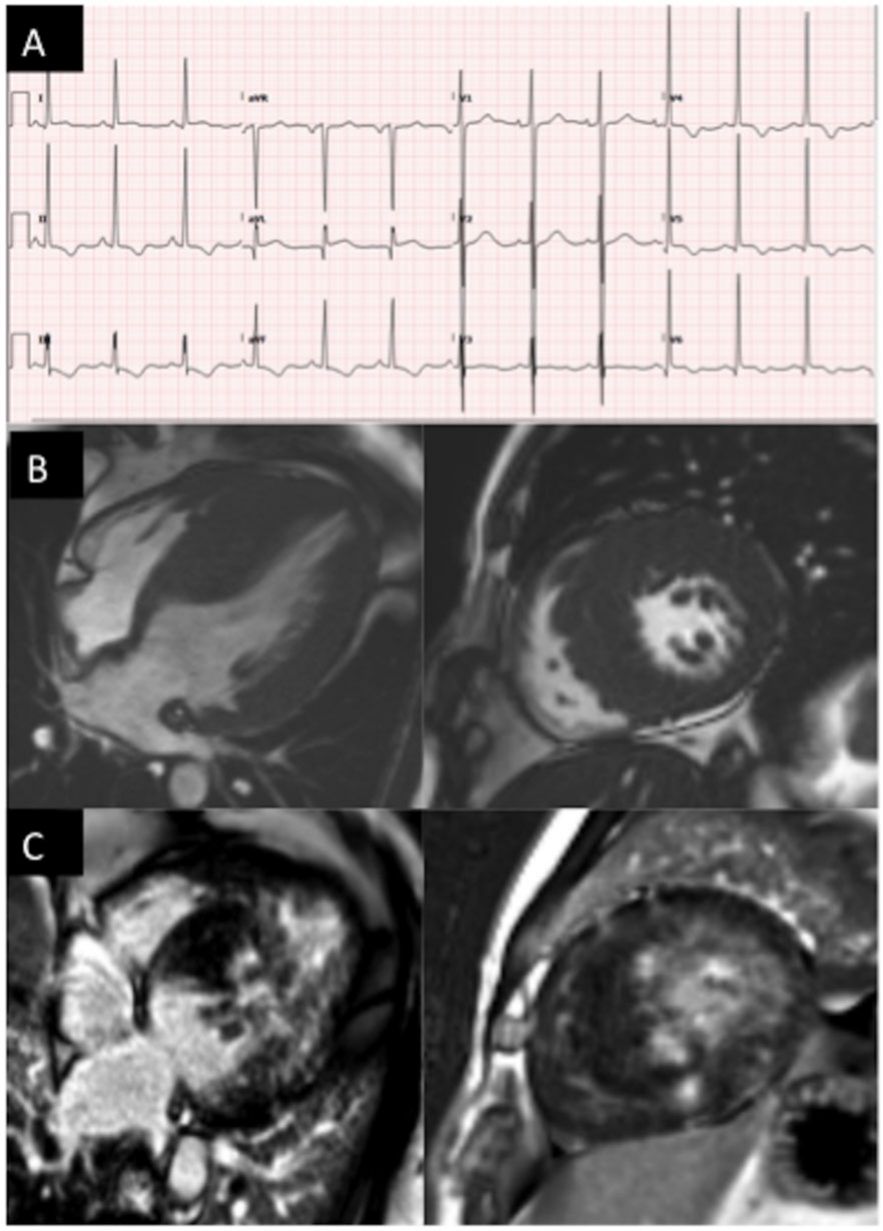
ECG (*A*), cine CMR image (*B*), and late gadolinium enhancement (*C*) images (four-chamber view on the left and mid short axis view on the right) of proband # 6 from the discovery cohort (*ALPK3* p.Gln1258*) illustrating some of the common phenotype traits of this cohort, including very high voltages on the ECG and extensive fibrosis. CMR, cardiovascular magnetic resonance; ANOVA, analysis of variance.

Six (12%) patients with *ALPK3*tv had resting left ventricular outflow tract obstruction and 3 of 49 (6%) had left ventricular systolic dysfunction (left ventricular ejection fraction <50%) at baseline evaluation; 3 patients developed left ventricular systolic dysfunction during follow-up. Eighteen (35%) HCM had >20 mm maximum left ventricular wall thickness and 16 (49%) had extensive myocardial fibrosis (assessed qualitatively as >15% of left ventricular segments affected) in 33 with cardiac magnetic resonance imaging. *Figure 5* and Supplementary material online, *Figure S1* show examples of a recurrent phenotype in this cohort.

#### Skeletal muscle involvement

Seven patients had raised serum creatine kinase out of 35 where it was determined (20%), one of whom was referred for skeletal muscle biopsy and had myopathic features and marked hypertrophic fibres with no evidence of inflammation (complete description in Supplementary material online, *Table S4*).

#### Outcomes

Follow-up time for patients with *ALPK3*tv and HCM was 5.3 ± 5.7 years. Fourteen patients (27.5%) were considered to be at high risk of SCD and were referred for an implantable cardioverter–defibrillator (ICD); two declined implant and no appropriate shocks have been registered during 4.0 ± 3.6 years of follow-up in the remainder. The patient with an ACM phenotype had an ICD implanted and had an appropriate shock. Four HCM patients (8%) were referred for cardiac transplantation, one of whom died of heart failure, one declined, one awaits and one was transplanted. One other patient died of heart failure.

**Figure 6:**
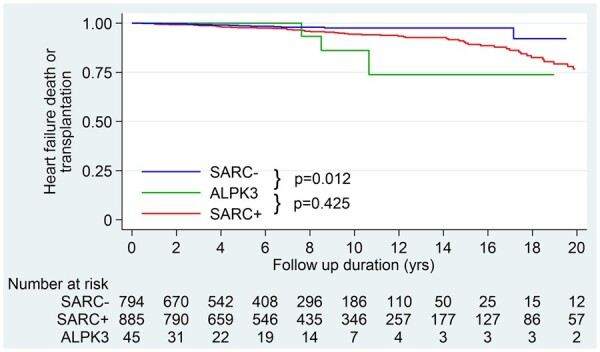
Kaplan–Meier analysis comparing incidence of an outcome of heart failure death and cardiac transplant between *ALP3*tv patients, sarcomere positive and sarcomere negative. Patients with ALPK3tv (probands and affected relatives from discovery and validation cohorts) with available follow-up data (45 out of 51) were compared to adult patients with HCM with available genotype and follow-up data.

A survival analysis for an outcome of heart failure death or transplant in patients with *ALPK3*tv showed a similar incidence compared to the sarcomere-positive population but significantly higher than in sarcomere-negative patients (*Figure 6*).

### Family co-segregation and disease penetrance

*ALPK3*tv co-segregated with HCM in all seven available families. Combined LOD score was 2.99, indicative of significant segregation (*P* < 0.05).^22^ The family pedigrees are represented in *Figure 1*.

The information on the entire cohort suggests that *ALPK3*tv are associated with incomplete penetrance until the age of 75 years (*Figure 2*), when the cumulative percentage of diagnosed carriers increased to >95% among males and 80% among females. A positive correlation was observed for age and left ventricular wall thickness in females but not in males (*Figure 3*).

### Histopathology

**Figure 7:**
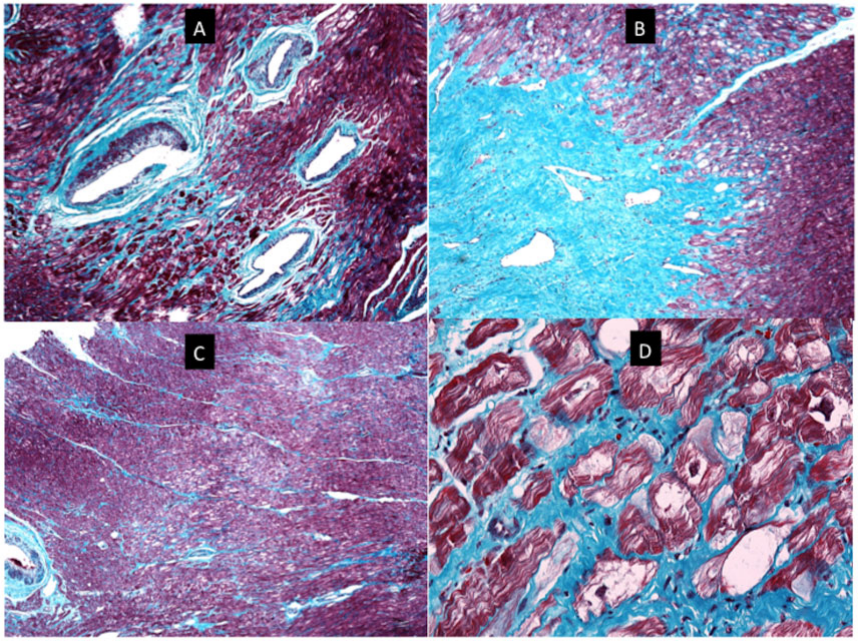
Histopathology images for index patient # 23. (*A*) A section of myocardium stained with Masson trichrome. There are four dysplastic vessels in the field. The muscle of the tunica media is irregularly distributed around the circumference of the vessels and in places is almost absent. There is accompanying mural fibrosis. The surrounding myocardium shows patchy interstitial fibrosis and focal myocyte vacuolation. (*B*) Low power view of myocardium showing a collagenous scar. The scar tissue contains thin-walled ectatic vessels and interdigitates with the surrounding myocardium that is vacuolated and shows foci of fine interstitial fibrosis (Masson-trichrome stain). (*C*) Low-power view of myocardium stained with Masson trichrome. The field shows an area of central pallor caused by a localized focus of vacuolated myocytes. There is fine interstitial fibrosis. There is no myocyte disarray. A dysplastic vessel is visible at the left edge of the field. (*D*) High-power view of a section of myocardium stained with Masson trichrome. It contains myocytes with irregular central areas of clearing of the cytoplasm to give vacuoles. Some of the vacuoles are traversed by fine strands of cytoplasm and other contain abundant normal mitochondria (seen as small red dots). Many of the vacuoles, however, are empty. Periodic acid-Schiff staining was negative. Desmin and plakoglobin staining was normal (Supplementary material online).

Histopathology analysis of the myocardial tissue in two probands (*Figure 7* and Supplementary material online, *Figures S2* and *S3*) showed cardiomyocyte hypertrophy with focal scarring and no significant cardiomyocyte disarray. There was endocardial fibrosis and the intramyocardial vessels were dysplastic. The cardiomyocytes were focally vacuolated in both patients around the areas of fibrosis, but vacuolation was also present in some non-fibrotic areas. Desmin and plakoglobin staining showed normal distribution patterns and intensities.

## Discussion

In this study, we show that truncating variants in *ALPK3* cause autosomal dominant HCM characterized by a severe cardiac phenotype with extensive myocardial fibrosis and progression to heart failure. Histopathological analysis of cardiac tissue confirmed extensive fibrosis and cardiomyocyte vacuolation with minimal myocyte disarray.

*ALPK3* is a poorly studied protein, but from the limited data available, it seems to be involved in the phosphorylation of cardiac relevant transcription factors including HEY2^23^ and in cardiomyocyte differentiation. Recent evidence has shown that *ALPK3* participates in intercalated disc and sarcomere structural organization and murine knock-out models show ventricular hypertrophy and impaired contractility.^3^ ^,^ ^24^ Abnormal calcium handling has been observed in cardiomyocytes differentiated from stem cells carrying homozygous *ALPK3* variants.^4^

Biallelic *ALPK3*tv were first identified in five children (four diagnosed *in utero*) presenting with severe, early-onset cardiomyopathy. Three died from heart failure between 35 weeks of gestation and 5 days after birth, and two survived up to the age of 11 years with severe concentric HCM.^3^ Similar phenotypes were reported in other small case series of biallelic *ALPK3*tv carriers.^5–7^ A recently expanded cohort of 19 patients with biallelic *ALPK3* variants, including 9 of the previously published paediatric patients (10 new probands including two compound heterozygous adults), displayed similar phenotypes, with most presenting initially as DCM without LVH and then evolving to HCM with impaired systolic function associated with extracardiac manifestations including scoliosis, facial dysmorphism and cleft palate.^8^ This series included compound heterozygotes with missense variants, for which pathogenicity is more challenging to ascertain. In our study, we did not analyse patients with only rare missense *ALPK3* variants.

We have identified *ALPK3*tv in 1.56% of HCM probands and confirmed significant enrichment compared to controls and gnomAD. This is in line with data reported in a recent publication, where an increased burden against gnomAD was reported for a Dutch population of 1548 index patients with various types of cardiomyopathy and a US proband cohort of 149 cardiomyopathy patients, including 129 with HCM (8 carrying *ALPK3*tv)^8^; however, the phenotypes of the heterozygous patients were not described in this report. Importantly, we have demonstrated co-segregation for *ALPK3*tv in heterozygosity.

All variants in our study were absent or very rare in gnomAD.^17^ p.Arg1059*, a stop-codon variant, was previously reported^3^ ^,^ ^8^ as a cause of autosomal recessive cardiomyopathy (p.Arg1261*, in the previous isoform version). Previously published variants also include the very recently reported p.Trp1563* (previously published in homozygosity, p.Trp1765* in the previous isoform), p.Pro804Alafs*4 (p.Pro1006fs*4 in the previous isoform), and p.Lys184* (p.Lys386*) in a US HCM cohort and p.Pro1385Leufs*23 (p.Pro1587Leufs*23) in a Dutch patient with ACM.^8^

Four variants showed a relatively high prevalence. One was the aforementioned p.Arg1059*, present in five of the patients in our cohort and another two truncating variants were present in 3 patients each.

Truncating variants were mainly stop codon and frameshift, with only 1 splice-site variant identified. *ALPK3*tv were mainly present in the largest exons 5 and 6, but were also additionally scattered throughout the gene, including distinct functional domains. No truncating variants were found in the last exon. These data suggest haploinsufficiency due to RNA nonsense mediated decay as the main mechanism of pathology.

To date, clinical descriptions of heterozygous carriers of *ALPK3*tv have shown variable findings with only 5 of 37 previously published heterozygous carriers fulfilling HCM criteria^8^ and a very recent case report describing co-segregation of a heterozygous *ALPK3*tv in one family.^14^ Evidence from our study points to possible incomplete (age-related) penetrance, which is quite usual in most autosomal dominant HCM genes, and might explain the variable penetrance in smaller studies.

This study suggests that the HCM phenotype associated with *ALPK3*tv is characterized by a high prevalence of apical and concentric patterns of LVH and a low prevalence of left ventricular outflow tract obstruction. Almost half of patients had extensive fibrosis on cardiac magnetic resonance. Although not directly comparable as we did not perform a quantitative assessment, this still seems to be a much higher proportion than is reported in large cohort studies, including the recently published HCM Registry study, where 9.1% had extensive scar.^25^ The extensive fibrosis was also confirmed in the histopathology analysis.

One-fourth of *ALPK3*tv carriers was judged to be at high risk of SCD and were referred for ICD implantation; however, no appropriate shocks were reported during a relatively short follow-up period. Thus, the arrhythmic risk profile associated with this phenotype remains to be ascertained, particularly in comparison with other genetic causes of autosomal dominant HCM.

Twelve percent of *ALPK3*tv carriers had left ventricular systolic dysfunction at baseline or during follow-up and almost 10% were referred for cardiac transplantation. The incidence of end-stage heart failure during follow-up was similar to a cohort of patients without a detectable pathogenic genetic variant, but survival analysis demonstrated a similar rate of heart failure endpoints when compared to carriers of pathogenic sarcomere gene mutations. While these data should be interpreted with caution as the *ALPK3*tv cohort was much smaller and the follow-up duration shorter than in the comparator populations, the higher prevalence of myocardial scar and frequent referral for transplantation suggests that the risk of progressive myocardial dysfunction is high in *ALPK3*tv carriers.

When stratifying the enrichment analysis for ethnicity in the discovery cohort, the OR for South Asians was 44.75 compared to 10.92 in whites. This observation must be confirmed in other cohorts, but it is of potential relevance as some non-white cohorts including South Asians tendentially show a larger prevalence of genotype elusive (VUS and genotype negative) patients,^26^ where the discovery of novel causal genes would have a significant clinical impact.

In our cohort, the phenotypes for the single homozygous patient and for the double heterozygotes with sarcomere variants did not seem substantially different from the heterozygous *ALPK3*tv patients. This is inconsistent with previous publications where homozygous patients were characterized by a severe form of cardiomyopathy with prominent extra-cardiac features and childhood onset.

It has been suggested that patients with HCM and no detectable pathogenic mutations in sarcomere genes are likely to have relatively benign disease caused by an oligogenic/polygenic predisposition under a higher influence of environmental modifiers^12^; the corollary is that the screening strategies in genotype-negative families can be less stringent. However, our demonstration that disease in patients without sarcomere mutations can be caused by penetrant monogenic variants with a prognosis that is at least similar to and possibly worse than that associated with sarcomere gene mutations, shows that this is an oversimplification. Relatives that carry heterozygous *ALPK3*tv variants should be followed-up in accordance with current guidelines for pathogenic sarcomere variant carriers.

Histological findings in cardiac tissue reported in two previous studies from homozygous patients included features such as focal cardiomyocyte hypertrophy, and subendocardial fibroelastosis.^4^ ^,^ ^8^ Immunohistochemistry in one publication showed disrupted plakoglobin and desmoplakin in cardiac tissue, suggesting a downstream effect of abnormal *ALPK3* function on desmosome structure;^3^ it was also suggested that the effect on desmosome might partially contribute to the arrhythmogenic trait observed. We have re-analysed the cardiac tissue from two of our patients that had a septal myectomy and also observed myocyte hypertrophy and fibrosis without prominent cardiomyocyte disarray. Unexpectedly, we observed PAS negative vacuoles (indicating absence of glycogen), the significance of which remains to be determined. Interestingly, we observed a high prevalence of short PR interval compared to other genotyped patients, which is considered a red-flag for some inborn errors of metabolism causing HCM.^1^ We did not observe an abnormal pattern of plakoglobin or desmin staining.

Only one of our patients had clinically apparent skeletal muscle involvement, but one-fifth of the proband cohort had raised plasma creatine kinase indicating sub-clinical skeletal muscle disease. This has not been described in other cohorts, where extra-cardiac findings in biallelic variant patients consisted mainly of dysmorphic features.^8^ Histopathological assessment of peripheral (quadriceps) muscle from a homozygous patient in a previous report showed normal findings in contrast to that of the heterozygous patient in our cohort, where myopathic features were described. It is possible that the genetic location of the variants might generate different phenotypes but more studies are required to clarify the relation between *ALPK3*tv and skeletal muscle involvement.

### Limitations

The cohort of patients with *ALPK3*tv had a relatively short follow-up, mostly reflecting the very recent application whole-exome sequencing strategies or the inclusion in large panels as a possible candidate gene. This resulted in a limited power to confirm the observed tendency for left ventricular systolic dysfunction and a higher number of heart failure-related events. The absence of arrhythmic events despite of the high proportion of ICD referrals, fibrosis and non-sustained ventricular tachycardia at baseline and follow-up did not allow modelling of SCD risk in patients with this particular genotype.

In common with other multicentre studies, the data collection is inevitably subject to a degree of heterogeneity. Particularly for a more accurate and quantitative comparison of fibrosis in the cardiac magnetic resonance imaging data between this cohort and other genotyped HCM patients, a central imaging laboratory analysis should be applied.

Previously, we have reported that *ALPK3*-deficient cardiomyocytes in homozygosity display altered contraction kinetics.^4^ Further studies on these models are essential to further refine the pathogenicity of rare *ALPK3* variants in heterozygosity.

## Conclusions

Heterozygous *ALPK3*tv are associated with an autosomal dominant form of HCM that is characterized by a distinctive clinical phenotype.

## Supplementary material

Supplementary material is available at *European Heart Journal* online. 

## Funding

Luis R Lopes is funded by a Medical Research Council (MRC) Clinical Academic Research Partnership (CARP) award (MR/T005181/1). The work at UCL was funded by the British Heart Foundation Program Grant (RG/15/8/31480) and the National Institute for Health Research University College London Hospitals Biomedical Research Centre. Other funding includes grant and fellowship support from National Health and Medical Research Council of Australia (E.R.P., D.A.E.), Australian Research Council (E.R.P.), Heart Foundation of Australia (E.R.P.), The Stafford Fox Medical Research Foundation (E.R.P., D.A.E.), and the Royal Children’s Hospital Foundation (D.A.-B., E.R.P., D.A.E.).

**Conflict of interest:** M.L. has received consultancy fees from Pfizer. A.R. is funded by a Sanofi-Genzyme research grant. D.Z. reports personal fees from Boehringer Ingelheim, AstraZeneca, KRKA, Takeda, Sanofi Aventis, Bayer, Servier, and Pfizer, outside the submitted work. J.P.-F. has received grants from Pfizer. M.O.-G. receives personal fees from Health in Code. S.G.-H. and L.d.l.H. are employees of Health in Code SL. L.M. is a stakeholder and CEO of Health in Code SL. P.M.E. has received consultancy and speaker fees from Pfizer, Sanofi Genzyme, DinQor, Sarepta, MyoKardia/Bristol Myers Squibb, Idorsia, and Astra Zeneca.

### Data availability

The data underlying this article will be shared on reasonable request to the corresponding author.

## Supplementary Material

ehab424_Supplementary_Data
