# Supplementary material for: Alpha-protein kinase 3 (ALPK3) truncating variants are a cause of autosomal dominant hypertrophic cardiomyopathy
Source: Eur Heart J. 2021 Jul 15;42(32):3063–73. doi: 10.1093/eurheartj/ehab424 (PMC8380059; doi:10.1093/eurheartj/ehab424)
Supplement: ehab424_Supplementary_Data [file ehab424_supplementary_data.zip › ehab424-suppl_data/Supplementarymaterial_EHJ_revised_12.5.2021_PL.docx]

**SUPPLEMENTARY METHODS**

**Enrichment analysis stratified for ethnicity – power calculation**

The enrichment analysis was performed using Fisher's exact test, designed for evaluating differences between two groups in terms of prevalence/frequencies even if the available number of elements under comparison is limited. The power when detecting the observed burden difference of 5.51% is 0.97 (above 80%).

**Comparison of HCM patients with ALPK3tv with the phenotype and outcome of a large genotyped control HCM cohort**

HCM was defined as a maximum left ventricular (LV) wall thickness ≥15mm unexplained solely by loading conditions or in accordance with published criteria for the diagnosis of disease in relatives of patients with unequivocal disease ^1^ . Patients with inherited metabolic diseases or syndromic causes of HCM were excluded.

Genetic testing focused on the 8 sarcomere genes definitively associated with HCM (*MYBPC3, MYH7, TNNT2, TNNI3, TPM1, MLY2, MYL3,* and *ACTC1*), and was performed using different platforms available over time. Variants were classified as pathogenic, likely pathogenic, unknown significance, or likely benign/benign using the current criteria of the American College of Medical Genetics and Genomics ^2^. Patients with ≥1 pathogenic or likely pathogenic variant were designated as sarcomeric-positives, while those with no pathogenic or likely pathogenic variants were designated as sarcomeric-negatives. Patients with ≥1 variants of unknown significance and no pathogenic or likely pathogenic variant were excluded from the analysis.

Left ventricular hypertrophy on ECG was defined according to the Sokolow-Lyon criteria (SV1+RV5/6>35mV). Left bundle branch block, right bundle branch block, and nonspecific intraventricular conduction delay were classified according to international criteria ^3^. Non-sustained ventricular tachycardia was defined as ≥3 consecutive ventricular beats at a rate of ≥120 bpm and <30s in duration on Holter monitoring (minimum duration 24 hours) at or prior to first evaluation ^4^.

Maximum left ventricular (LV) wall thickness was defined as the greatest thickness measured in the parasternal short-axis plane using 2-D echocardiography ^5^. LV ejection fraction was calculated by Simpson’s biplane or Teichholz method. The left atrial diameter was determined by M-Mode or 2D echocardiography in the parasternal long axis plane.

The cause of death was ascertained by experienced cardiologists using hospital and primary health care records, death certificates, post-mortem reports, and interviews with witnesses (relatives and physicians). The survival analysis was based on a composite endpoint consisting of heart-failure death and heart transplantation. The follow-up time for each patient was taken to be the time from first evaluation at our centre to the composite endpoint, death or last follow-up date. Patients who were alive at the last evaluation or died with a cause of death other than heart failure were treated as censored.

**SUPPLEMENTARY RESULTS**

**Comparison of the burden of rare synonymous variants**

The prevalence of rare synonymous *ALPK3* variants was similar among these three cohorts:

Discovery cohort *vs* gnomAD: 8 out of 770 vs 730 out of 151783 /2: OR 1.08 (CI 95%, 0.46-2.15). p value=0.7116

Validation cohort *vs* gnomAD: 18 out of 2047 vs 730 out of 151783/2: OR 0.91 (CI 95%, 0.53-1.46). p value=0.8181

Validation cohort *vs* discovery cohort: 18 out of 2047 vs 18 out of 770: OR 0.85 (CI 95%, 0.35-2.26). p value=0.663

**Lack of enrichment for** **dilated cardiomyopathy**

1 case out of 746 cases with this phenotype vs 74 alleles in gnomAD, and 150862/2 individuals, OR 1.36 (IC, 95%, 0.03-7.87, p-value = 0.5223)

**Supplementary tables**

**Supplementary table 1.** List of 261 genes sequenced in the validation cohort.

| **Gene** | **Protein name** |
| --- | --- |
| *A2ML1* | alpha-2-macroglobulin-like protein 1 |
| *AARS2* | Alanine--tRNA ligase, mitochondrial |
| *ABCC9* | ATP-binding cassette, sub-family C (CFTR/MRP), member 9 |
| *ACAD9* | Acyl-CoA dehydrogenase family member 9, mitochondrial |
| *ACADVL* | Very long-chain specific acyl-CoA dehydrogenase, mitochondrial |
| *ACTA1* | Actin, alfa 1, skeletal muscle |
| *ACTC1* | Actin, alpha cardiac muscle 1 |
| *ACTN2* | Alpha-actinin-2 |
| *AGK* | Acylglycerol kinase, mitochondrial |
| *AGL* | Glycogen debranching enzyme |
| *AGPAT2* | 1-acyl-sn-glycerol-3-phosphate acyltransferase beta |
| *AKAP9* | A-kinase anchor protein 9 |
| *AKT1* | RAC-alpha serine/threonine-protein kinase |
| *ALMS1* | Alstrom syndrome protein 1 |
| *ALPK3* | alpha-protein kinase 3 |
| *ANK2* | Ankyrin 2 |
| *ANK3* | Ankyrin-3 |
| *ANKRD1* | Ankyrin repeat domain-containing protein 1 |
| *ANO5* | Anoctamin-5 |
| *ATP5F1E* | ATP synthase subunit epsilon, mitochondrial |
| *ATPAF2* | ATP synthase mitochondrial F1 complex assembly factor 2 |
| *BAG3* | BAG family molecular chaperone regulator 3 |
| *BRAF* | Serine/threonine-protein kinase B-raf |
| *BSCL2* | Seipin |
| *C10orf71* | Cardiac enriched FHL2-interacting protein |
| *CACNA1C* | Voltage-dependent L-type calcium channel subunit alpha-1C |
| *CACNA1D* | Voltage-dependent L-type calcium channel subunit alpha-1D |
| *CACNA2D1* | Voltage-dependent calcium channel subunit alpha-2/delta-1 |
| *CACNB2* | Voltage-dependent L-type calcium channel subunit beta-2 |
| *CALM1* | Calmodulin |
| *CALM2* | Calmodulin |
| *CALM3* | Calmodulin |
| *CALR* | calreticulin |
| *CALR3* | Calreticulin 3 |
| *CAPN3* | Calpain-3 |
| *CASQ2* | Calsequestrin-2 |
| *CASZ1* | zinc finger protein castor homolog 1 |
| *CAV3* | Caveolin 3 |
| *CAVIN1* | Polymerase I and transcript release factor |
| *CAVIN4* | Caveolae-associated protein 4 |
| *CBL* | E3 ubiquitin-protein ligase CBL |
| *CDH2* | cadherin-2 |
| *CHRM2* | muscarinic acetylcholine receptor M2 |
| *COA5* | cytochrome c oxidase assembly factor 5 |
| *COA6* | cytochrome c oxidase assembly factor 6 homolog |
| *COL7A1* | collagen alpha-1(VII) chain |
| *COQ2* | 4-hydroxybenzoate polyprenyltransferase, mitochondrial |
| *COX15* | Cytochrome c oxidase assembly protein COX15 homolog |
| *COX6B1* | Cytochrome c oxidase subunit 6B1 |
| *CRYAB* | Alpha-crystallin B chain |
| *CSNK1A1* |  |
| *CSRP3* | Cysteine and glycine-rich protein 3 |
| *CTNNA1* | Catenin alpha-1 |
| *CTNNA3* | catenin alpha-3 |
| *CTNNB1* | catenin beta-1 |
| *DES* | Desmin |
| *DLD* | Dihydrolipoyl dehydrogenase, mitochondrial |
| *DMD* | Dystrophin |
| *DNAJC19* | Mitochondrial import inner membrane translocase subunit TIM14 |
| *DNM1L* | Dynamin-1-like protein |
| *DOLK* | Dolichol kinase |
| *DSC2* | Desmocollin 2 |
| *DSG2* | Desmoglein 2 |
| *DSP* | Desmoplakin |
| *DTNA* | Dystrobrevin alpha |
| *ELAC2* | Zinc phosphodiesterase ELAC protein 2 |
| *EMD* | Emerin |
| *EYA4* | Eyes absent homolog 4 |
| *FAH* | Fumarylacetoacetase |
| *FBXO32* | F-box only protein 32 |
| *FGF12* | Fibroblast growth factor 12 |
| *FHL1* | Four and a half LIM domains protein 1 |
| *FHL2* | Four and a half LIM domains 2 (FHL-2) |
| *FHOD3* | FH1/FH2 domain-containing protein 3 |
| *FKRP* | Fukutin-related protein |
| *FKTN* | Fukutin |
| *FLNC* | Filamin-C |
| *FOXD4* | Forkhead box protein D4 |
| *FOXRED1* | FAD-dependent oxidoreductase domain-containing protein 1 |
| *FXN* | Frataxin, mitochondrial |
| *GAA* | Lysosomal alpha-glucosidase |
| *GATA4* | Transcription factor GATA-4 |
| *GATA5* | Transcription factor GATA-5 |
| *GATA6* | Transcription factor GATA-6 |
| *GATAD1* | GATA zinc finger domain-containing protein 1 |
| *GFM1* | Elongation factor G, mitochondrial |
| *GJA1* | Gap junction alpha-1 protein |
| *GJA5* | Gap junction alpha-5 protein |
| *GLA* | Alpha galactosidase A |
| *GLB1* | Beta-galactosidase |
| *GNB2* | guanine nucleotide-binding protein G(I)/G(S)/G(T) subunit beta-2 |
| *GNPTAB* | N-acetylglucosamine-1-phosphotransferase subunits alpha/beta |
| *GPD1L* | Glycerol-3-phospate dehydrogenase 1-like protein |
| *GREM2* | Gremlin-2 |
| *GSK3B* | glycogen synthase kinase-3 beta |
| *GUSB* | Beta-glucuronidase |
| *GYG1* | glycogenin-1 |
| *HCN4* | Potassium/sodium hyperpolarization-activated cyclic nucleotide-gated channel 4 |
| *HFE* | Hereditary hemochromatosis protein |
| *HRAS* | GTPase HRas |
| *IDH2* | isocitrate dehydrogenase (NADP), mitochondrial |
| *ILK* | Integrin-linked protein kinase |
| *IRX3* | iroquois-class homeodomain protein IRX-3 |
| *ISM2* | Isthmin-2 |
| *JARID2* | Jumonji (Jmj) A/T-rich interaction domain 2 |
| *JPH2* | Junctophilin 2 |
| *JUP* | Junction plakoglobin |
| *KAT6B* | Histone acetyltransferase KAT6B |
| *KCNA5* | Potassium voltage-gated channel subfamily A member 5 |
| *KCND2* | Potassium voltage-gated channel subfamily D member 2 |
| *KCND3* | Potassium voltage-gated channel subfamily D member 3 |
| *KCNE1* | Potassium voltage-gated channel subfamily E member 1 |
| *KCNE2* | Potassium voltage-gated channel subfamily E member 2 |
| *KCNE3* | Potassium voltage-gated channel subfamily E member 3 |
| *KCNE5* | Potassium voltage-gated channel subfamily E member 1-like protein |
| *KCNH2* | Potassium voltage-gated channel subfamily H member 2 |
| *KCNJ2* | Inward rectifier potassium channel 2 |
| *KCNJ5* | G protein-activated inward rectifier potassium channel 4 |
| *KCNJ8* | ATP-sensitive inward rectifier potassium channel 8 |
| *KCNK17* | Potassium channel subfamily K member 17 |
| *KCNK3* | potassium channel subfamily K member 3 |
| *KCNQ1* | Potassium voltage-gated channel subfamily KQT member 1 |
| *KLF10* | Krueppel-like factor 10 |
| *KLHL24* | kelch-like protein 24 |
| *KRAS* | GTPase KRas |
| *LAMA2* | Laminin subunit alpha-2 |
| *LAMA4* | Laminin subunit alpha-4 |
| *LAMP2* | Lysosome-associated membrane glycoprotein 2 |
| *LDB3* | LIM domain-binding protein 3 |
| *LDLR* | Low density lipoprotein receptor |
| *LIAS* | Lipoyl synthase, mitochondrial |
| *LMNA* | Prelamin-A/C |
| *LMOD2* | Leiomodin-2 |
| *LZTR1* | Leucine-zipper-like transcriptional regulator 1 |
| *MAP2K1* | Dual specificity mitogen-activated protein kinase kinase 1 |
| *MAP2K2* | Dual specificity mitogen-activated protein kinase kinase 2 |
| *MAP3K8* | Mitogen-activated protein kinase kinase kinase 8 |
| *MEF2C* | Myocyte-specific enhancer factor 2C |
| *MIB1* | E3 ubiquitin-protein ligase MIB1 |
| *MIR208A* |  |
| *MIR208B* |  |
| *MLYCD* | Malonyl-CoA decarboxylase, mitochondrial |
| *MRPL3* | 39S ribosomal protein L3, mitochondrial |
| *MRPL44* | 39S ribosomal protein L44, mitochondrial |
| *MRPS22* | 28S ribosomal protein S22, mitochondrial |
| *MTO1* | Protein MTO1 homolog, mitochondrial |
| *MYBPC3* | Myosin-binding protein C, cardiac-type |
| *MYBPHL* | myosin-binding protein H-like |
| *MYH11* | Myosin-11 |
| *MYH6* | Myosin-6 |
| *MYH7* | Myosin-7 |
| *MYL2* | Myosin regulatory light chain 2, ventricular/cardiac muscle isoform |
| *MYL3* | Myosin light chain 3 |
| *MYLK2* | Myosin light chain kinase 2, skeletal/cardiac muscle |
| *MYOM1* | Myomesin-1 |
| *MYOT* | Myotilin |
| *MYOZ2* | Myozenin-2 |
| *MYPN* | Myopalladin |
| *NEBL* | Nebulette |
| *NEXN* | Nexilin |
| *NF1* | Neurofibromin |
| *NKX2-5* | Homeobox protein Nkx-2.5 |
| *NKX2-6* | Homeobox protein Nkx-2.6 |
| *NNT* | NAD(P) transhydrogenase, mitochondrial |
| *NONO* | Non-POU domain-containing octamer-binding protein |
| *NOS1AP* | Carboxyl-terminal PDZ ligand of neuronal nitric oxide synthase protein |
| *NOTCH1* | Neurogenic locus notch homolog protein 1 |
| *NPPA* | Atrial natriuretic factor |
| *NRAP* | Nebulin-related anchoring protein |
| *NRAS* | GTPase NRas |
| *OBSCN* | Obscurin |
| *OBSL1* | Obscurin-like protein 1 |
| *OPA3* | optic atrophy 3 protein |
| *PDHA1* | Pyruvate dehydrogenase E1 component subunit alpha, somatic form, mitochondrial |
| *PDLIM3* | PDZ and LIM domain protein 3 |
| *PERP* | p53 apoptosis effector related to PMP-22 |
| *PHKA1* | Phosphorylase b kinase regulatory subunit alpha, skeletal muscle isoform |
| *PITX2* | Pituitary homeobox 2 |
| *PKD2* | Polycystin-2 |
| *PKP2* | Plakophilin 2 |
| *PKP4* | Plakophilin 4 |
| *PLN* | Cardiac phospholamban |
| *PMM2* | Phosphomannomutase 2 |
| *PPA2* | Inorganic pyrophosphatase 2, mitochondrial |
| *PPCS* | phosphopantothenate--cysteine ligase |
| *PPP1CB* | serine/threonine-protein phosphatase PP1-beta catalytic subunit |
| *PPP1R13L* | relA-associated inhibitor |
| *PRDM16* | PR domain zinc finger protein 16 |
| *PRKAG2* | 5'-AMP-activated protein kinase subunit gamma-2 |
| *PSEN1* | Presenilin-1 |
| *PSEN2* | Presenilin-2 |
| *PTPN11* | Tyrosine-protein phosphatase non-receptor type 11 |
| *QRSL1* | Glutamyl-tRNA(Gln) amidotransferase subunit A, mitochondrial |
| *RAF1* | RAF proto-oncogene serine/threonine-protein kinase |
| *RANGRF* | Ran guanine nucleotide release factor |
| *RASA1* | ras GTPase-activating protein 1 |
| *RASA2* | ras GTPase-activating protein 2 |
| *RBM20* | RNA-binding motif protein 20 |
| *RBM24* | RNA-binding protein 24 |
| *RIT1* | GTP-binding protein Rit1 |

**Supplementary table 2.** Logarithm of the odds (LOD) score for *ALPK3*tv and HCM families.

| Pedigree | ***ALPK3* variant** | Number of Individuals genotyped | Number of carriers | LOD score 95% | LOD score 80% |
| --- | --- | --- | --- | --- | --- |
| A | **p.Glu1146Glyfs*12** | 5 | 4 | *0.0775649* | *0.0773757* |
| B | **p.Trp1563*** | 5 | 3 | *0.5771392* | *0.5181878* |
| C | **p.Glu1179Argfs*93** | 2 | 2 | 0 | 0 |
| D | **p.Pro45Alafs*37** | 9 | 3 | *1.101545* | *0.8727593* |
| E |  | 5 | 4 | *0.5963732* | *0.5944988* |
| F | **p.Glu1098*** | 7 | 2 | *0.5547764* | *0.4388721* |
| G | **p.Lys184*** | 2 | 2 | *0.07791167* | *0.07771273* |
| TOTAL LODs SCORE | | | | ***2.985311*** | ***2.579406*** |

LOD score 95%: Logarithm of the odds score calculated for a disease penetrance of 95%.

LOD score 80%: Logarithm of the odds score calculated for a disease penetrance of 80%.

**Supplementary table 3.** Baseline phenotype and outcomes of the probands.

|  | **ECG** | **Echocardiogram** | **Cardiac MRI** | **Creatine kinase** | **Holter – AF or NSVT (baseline or follow-up)** | **ICD** | **ASA/Myectomy** | **Transplant/**  **Mortality** | **Histology** |
| --- | --- | --- | --- | --- | --- | --- | --- | --- | --- |
| **Discovery cohort - London** |  |  |  |  |  |  |  |  |  |
| 1 | SR, inferior and lateral TWI | ASH, MLVWT 15 mm apical septum, no LVOTO, LVEF 75% | ASH, MLVWT 22mm, extensive LGE | Normal | NSVT | No (but lost to follow-up) | No | No |  |
| 2 | SR, LVH, ST elevation lateral/left precordial leads | ASH, MLVWT 30mm, no LVOTO, LVEF 77% | ASH, MLVWT 26mm, extensive LGE | Normal | NSVT | Yes | No | No |  |
| 3 | SR, LVH, LAHB, lateral TWI | ASH, MLVWT 19mm, no LVOTO, LVEF 74 % | ASH, MLVWT 25mm, extensive LGE | Normal | No | No | No | No |  |
| 4 | AF, LAHB, poor R wave progression, abnormal intraventricular conduction, Q wave lateral leads | ASH, MLVWT 13mm, no LVOTO, low LVEF 50%, impaired longitudinal function, restrictive | ASH, MLVWT16 mm, LV impairment, extensive LGE | Raised (200) | AF, NSVT | Referred - declined | No | HF death 2017 |  |
| 5 | SR, LVH, inferior and lateral TWI | Apical, MLVWT 18mm, no LVOTO, LVEF 61% | Apical, MLVWT 15mm, mild LGE apex | Raised (459) | No | No | No | No |  |
| 6 | SR, LVH (very high voltages), widespread TWI | ASH, MLVWT 21mm, mid-cavity obstruction, no LVOTO, impaired longitudinal function, LVEF 70% | ASH, MLVWT 26mm, extensive LGE | Raised (615) | NSVT | Referred - declined | No | No | Peripheral muscle biopsy: Myopathic features, marked hypertrophic fibres, dystrophy panel no abnormality, few regenerating fibres, no evidence of a significant inflammation on H&E and no upregulation of MHC Class I at the periphery of fibres. |
| 7 | SR, LVH, inferior and lateral TWI | Symmetric LVH, MLVWT 21 mm increased towards apex, no LVOTO, restrictive | NA | Raised (1360) | AF, NSVT | Referred - declined | No | Referred for transplant-declined |  |
| 8 | SR, ST elevation inferior and left precordial | ASH, MLVWT 24mm, apical aneurysm, no LVOTO, LVEF 67% | ASH, MLVWT 20mm, extensive apical LGE, apical aneurysm | Normal | NSVT | No | No | No | Post-myectomy: figure 7 and main text for description |
| 9 | ST, LVH, left and lateral TWI | Apical, MLVWT 17mm, no LVOTO, impaired longitudinal function, LVEF 70%, restrictive | ASH-apical, MLVWT 32mm, impaired longitudinal, LVEF 63%, LGE NA | Normal | AF | No | No | No |  |
| 10 | SR, LVH, very high voltages, deep inverted TWI lateral leads | ASH, MLVWT 14mm, no LVOTO, LVEF 65% | ASH, MLVWT 17mm, patchy mid to apical septum LGE | NA | NSVT | Yes | No | No |  |
| 11 | SR, LVH, lateral and left TWI | Apical, MLVWT 16mm, no LVOTO, LVEF 65% | Apical, MLVWT 14mm, minimal LGE | Normal | NSVT | No | No | No |  |
| 12 | SR, inferolateral Q waves, predominant R wave right precordial leads | ASH, MLVWT 15mm, no LVOTO, LVEF 59% | ASH, MLVWT 16mm, extensive LGE | Normal | No | No | No | No |  |
| **International validation cohort** |  |  |  |  |  |  |  | No |  |
| 13 | SR, LVH, TWI left precordial and lateral | Apical LVH, mid-cavity 60mmHg | Mid-apical septal and apical, MLVWT 20mm, extensive and patchy LGE | NA | No | No | No | No |  |
| 14 | SR, LVH, TWI left precordial and lateral | ASH, MLVWT 14mm, no LVOTO, mild AS | ASH, MLVWT 18mm, no LGE | Raised (3155) | No | No | No | No |  |
| 15 | SR, LVH, LBBB-like, TWI left precordial and lateral | ASH, MLVWT 23mm, SAM with 75mmHg LVOTO | ASH, MLVWT 28mm, focal patchy LGE septum, LVOTO and SAM | Normal | NSVT, paroxysmal AF | Yes | No | No |  |
| 16 | SR, LVH/RVH, RBBB, LAHB | Concentric, MLVWT 15mm, no LVOTO | Mild ASH, 14mm, no LGE | Raised (1319) | NSVT | Yes | No | No |  |
| 17 | SR, LVH, TWI left precordial and lateral | ASH and apical, MLVWT 23mm, no LVOTO | Apical predominant, MLVWT 20mm, extensive LGE, apical aneurysm | Normal | NSVT | Yes | No | No |  |
| 18 | SR, LVH, very high voltages, TWI left precordial and lateral | Apical, MLVWT 12mm, no LVOTO | Apical, MLVWT 16 mm, apical LGE | Normal | No | No | No | No |  |
| 19 | SR, LVH, TWI lateral and left precordial | Apical, MLVWT 20mm, no LVOTO | NA | Raised (320) | No (AF during ETT) | No | No | No |  |
| 20 | SR, LVH, LBBB | Apical, MLVWT 20mm, no LVOTO | NA | Normal | No | No | No | No |  |
| 21 | SR, short PR, LVH, IRBBB, TWI | Apical, MLVWT 21mm, no LVOTO | NA | Normal | AF | No | No | No |  |
| 22 | SR, LVH, TWI lateral leads | ASH, MLVWT 14mm, no LVOTO | NA | NA | No | No | No | No |  |
| 23 | SR, LVH, very high voltages, TWI (“giant”), lateral and left precordial | ASH reverse, MLVWT 32mm, LVOTO 101 mmHg | ASH reverse, MLVWT 37 mm, extensive LGE | Normal | NSVT | No | Myectomy | No | Post-myectomy: figure 7 and main text for description |
| 24 | SR, short PR, LVH, TWI | ASH, MLVWT 21mm, LVOTO 33mmHg | ASH, MLVWT 19mm, extensive LGE | Normal | No | No | No | No |  |
| 25 | 1^st^ degree AVB, LVH, TWI | Apical, MLVWT 15mm, no LVOTO | Apical, MLVWT 15mm, no LGE | Normal | AF | No | No | No |  |
| 26 | SR, LVH, TWI III and aVF | Concentric, MLVWT 13 mm, no LVOTO | ASH, MLVWT 15 mm, LGE insertion point only, multiple clefts/crypts | NA | No | No | No | No |  |
| 27 | AF, LAHB, IRBBB | Concentric, MLVWT 25mm, LVEF 47%, no LVOTO | Concentric, MLVWT 25mm, LVEF 54%, RVH, circumferential LGE | Normal | AF | No | No | No |  |
| 28 | SR, LVH, LAHB, Q waves left precordial and lateral | HCM with aneurysm and impaired LV function, “burnout”, MLVWT 14mm, LVEF 30%, no LVOTO | Concentric, MLVWT 18mm, extensive LGE | NA | No | No | No | Waiting transplant |  |
| 29 | AF, LAHB | Concentric, MLVWT 15mm, no LVOTO | Concentric, patchy diffuse LGE | Normal | AF, sustained VT at presentation | No | No | No |  |
| 30 | SR, PR 200ms, RBBB, LVH, TWI V1 and V2, tall biphasic R/S complexes in several leads, prolonged QTc | Concentric, MLVWT 14mm, no LVOTO, grade II diastolic dysfunction | Concentric, MLVWT 14mm, RV hypertrophy, no LGE | Normal | No | No | No | No |  |
| 31 | SR, LVH, TWI all precordial | Concentric, MLVWT 19mm, no LVOTO, diastolic dysfunction | Concentric, MLVWT 18mm, no LGE | Normal | NSVT, AF | No | No | No |  |
| 32 | SR, LVH, TWI all precordial and inferior | ASH, MLVWT 17 mm, no LVOTO, diastolic dysfunction | NA | Normal | No | No | No | No |  |
| 33 | SR, PR 200ms, intraventricular conduction delay, LAHB, LVH, TWI anterolateral, pathological Q V1, V2 | SH, MLVWT 23mm, LVOTO 85mmHg | NA | Normal | NSVT | Yes | ASA | No |  |
| 34 | SR, intraventricular conduction delay, TWI anterolateral | Concentric, MLVWT 15mm | NA | NA | No | Yes | No | No | Post-myectomy: myocyte hypertrophy and mild interstitial fibrosis, no dissarray. |
| 35 | SR, LVH, TWI widespread | Concentric, MLVWT 16mm, no LVOTO | LVEF 60%; LGE 24% MLVWT 25mm | NA | NSVT | Yes | No | No |  |
| 36 | SR, LAHB, LVH, TWI lateral | ASH reverse, MLVWT 20mm, no LVOTO | ASH reverse, MLVWT 19mm, LGE mid-apical anterior | NA | No | No | No | No |  |
| 37 | SR, LVH, TWI left precordial | Apical, MLVWT 15mm, no LVOTO, mod diastolic dysfunction | Apical, 18 mm MLVWT, LGE apical | Normal | No | No | No | No |  |
| 38 | SR | ASH reverse, MLVWT 17mm, LVOTO 150mmHg, moderate diastolic dysfunction | ASH reverse, MLVWT 18mm, LGE midwall septum | Normal | No | No | No | No |  |
| 39 | SR, QS V3-V6, DII, DIII, aVF and J point elevation all these leads | ASH, MLVWT 16mm, LVEF 30% | ASH, LVEF 37%, extensive fibrosis (septal, inferior, apical) | NA | No | Yes CRT-D | No | No |  |
| 40 | SR, TWI anterior | ASH, MLVWT 16mm | NA | NA | NSVT | No | No | No |  |
| 41 | SR | LV dysfunction (no more details available) | NA | NA | No | No | No | Transplant 2008 |  |
| 42- ACM | SR, pathological Q waves inferior leads | AC phenotype, RV dilatation and impairment, LVEF 66% | LVEF 49%, RVEF 32%, LGE anterior LV and inferior RV | NA | NSVT | Yes - appropriate shock | - | No |  |
| 43 | SR, LVH, TWI left precordial and lateral | No LVH detected | Apical MLVWT 22mm, midwall LGE | Normal | NSVT | No | No | No |  |

Abbreviations: AF: atrial fibrillation; ASH: asymmetrical septal hypertrophy; ASA: alcohol septal ablation; CRT-D: cardiac resynchronization therapy with defibrillator; ETT: exercise treadmill test; HF: heart failure; ICD: implantable cardioverter defibrillator; LA: left atria; LAHB: left anterior hemiblock; LBBB: left bundle branch block; LGE: late gadolinium enhancement; LV: left ventricle; LVEF: left ventricular ejection fraction; LVH: left ventricular hypertrophy; LVOTO: left ventricular outflow tract obstruction; MLVWT: maximal left ventricular wall thickness; MRI: Magnetic resonance imaging; NSVT: non-sustained ventricular tachycardia; NA: not available; RBBB: right bundle branch block; RV: right ventricle; RVH: right ventricular hypertrophy; SCD: sudden cardiac death; SR: sinus rhythm; TWI: T wave inversion.

**Supplementary table 4.** Demographics, family history and genetic characteristics of the probands.

| **Patient n.** | **Age diagnosis** | **Sex (M: male, F: female)** | **Ethnicity** | ***ALPK3* Variant** (according to the isoform version: NM_020778.5/ NP_065829.4) | **GnomAD**  **MAF**  **(v3.1.1)** | **GnomAD**  **MAF,**  **matched for ethnicity** | **ClinVar** | **Sarcomere or other potentially causal variants** | **Family history of HCM or SCD** |
| --- | --- | --- | --- | --- | --- | --- | --- | --- | --- |
| **Discovery cohort - London** |  |  |  |  |  |  |  |  |  |
| 1 | 49 | M | White | p.Gln652*  c.1954C>T | 0.0000 | 0.0000 | - | *MYBPC3* VUS (p.Arg470Trp) |  |
| 2 | 46 | M | South Asian | p.Leu437Argfs*34  c.1310delT | 0.0000 | 0.0000 | - | No | SCD father and maternal 2^nd^ degree cousin |
| 3 | 46 | M | South Asian | p.Glu203*  c.607G>T | 0.0000 | 0.0000 | - | No | HCM brother |
| 4 | 61 | F | White | p.Asp1077Glufs*13  c.3231_3232delCG | 0.0000 | 0.0000 | - | No |  |
| 5 | 38 | M | White | p.Ser1192Profs*80  c.3574delT | 0.0000 | 0.0000 | - | No | HCM father |
| 6 | 39 | M | South Asian | p.Gln1258*  c.3772C>T | 0.0000 | 0.0000 | - | No | SCD uncle and cousin (uncle’s son) |
| 7 | 55 | M | South Asian | p.Arg1590*  c.4768C>T | 0.0000 | 0.0000 | Likely pathogenic | No |  |
| 8 | 72 | F | White | p.Arg1059*  c.3175C>T | 3.94x10^-5^ | 7.35 x10^-5^ | Conflicting interpretations of pathogenicity | No |  |
| 9 | 68 | F | Not stated | c.1654-1G>A (predicted fs) | 6.57x10^-6^ | - | - | No |  |
| 10 | 27 | M | White | p.Ala749Serfs*6  c.2244-2245insA | 0.0000 | 0.0000 | - | *MYH7* VUS (p.Ala1332Thr) |  |
| 11 | 32 | M | White | p.Pro769Thrfs*8  c.2304-2301insA | 0.0000 | 0.0000 | - | *MYBPC3* VUS (p.Arg215Cys) | SCD father |
| 12 | 72 | M | South Asian | p.Ile149Profs*25  c.442_443insCG | 0.0000 | 0.0000 | - | No |  |
| **International validation cohort** |  |  |  |  |  |  |  |  |  |
| 13 | 73 | F | White | p.Arg1059*  c.3175C>T | 3.94x10^-5^ | 7.35 x10^-5^ | Conflicting interpretations of pathogenicity | No |  |
| 14 | 65 | M | Not stated | p.Arg1059*  c.3175C>T | 3.94x10^-5^ | - | Conflicting interpretations of pathogenicity | No |  |
| 15 | 41 | M | White | p.Asp1077Glufs*13  c.3231_3232delCG | 0.0000 | 0.0000 | - | No |  |
| 16 | 52 | M | Black | p.Glu1364Valfs*11  c.4089_4093+12delTGAGGGTGAGTGTGCCC | 0.0000 | 0.0000 | - | No |  |
| 17 | 47 | M | White | p.Pro1385Leufs*23  c.4154delC | 6.58x10^-6^ | 1.47 x10^-5^ | - | *MYBPC3* VUS (p.Pro677Ser) |  |
| 18 | 33 | M | White | p.Pro45Alafs*37  c.128_131insTGCG | 0.0000 | 0.0000 | - | No | DCM and ICD father (primary prophylaxis) |
| 19 | 56 | M | White | p.?  c.-4_17delinsAGGG (start lost; 5 ´UTR) | 0.0000 | 0.0000 | - | No | LVH brother |
| 20 | 75 | F | White | p.Gln687*  c.2059C>T | 0.0000 | 0.0000 |  | No |  |
| 21 | 68 | F | White | p.Val813Glyfs*66  c.2437insG | 0.0000 | 0.0000 | - | No | SCD nephew |
| 22 | 37 | M | White | p.Arg1059*  c.3175C>T | 3.94x10^-5^ | 7.35 x10^-5^ | Conflicting interpretations of pathogenicity | No |  |
| 23 | 38 | M | White | p.Trp1563*  c.4689G>A | 0.0000 | 0.0000 | - | No | HCM father and sister |
| 24 | 57 | F | White | p.Glu1179Argfs*93  c.3535delG | 6.58x10^-6^ | 1.47 x10^-5^ | - | No | HCM father |
| 25 | 70 | F | White | p.Glu1179Argfs*93  c.3535delG | 6.58x10^-6^ | 1.47 x10^-5^ | - | No |  |
| 26 | 45 | M | White | p.Gly534Trpfs*37  c.1599insT | 0.0000 | 0.0000 | - | No |  |
| 27 | 80 | M | White | p.Ala335Profs*80  c.1003_1006delGCAG | 0.0000 | 0.0000 | - | No |  |
| 28 | 30 | F | White | p.Thr246Serfs*25  c.718_731insAGGGAGCCTGAGGG | 0.0000 | 0.0000 | - | No |  |
| 29 | 60 | M | White | p.Arg597*  c.1789A>T | 0.0000 | 0.0000 | - | No |  |
| 30 | 15 | F | White | p.Val847Alafs*12  c.2540_2549delinsCGGCCAGGGGGA | 0.0000 | 0.0000 | - | *FLNC* P (p.Gly2011Arg) | HCM and HF death father. HF death grandfather |
| 31 | 67 | M | White | p.Pro804Alafs*4  c.2408-2409insA | 0.0000 | 0.0000 | - | No |  |
| 32 | 43 | M | White | p.Pro45Alafs*37  c.128_131insTGCG | 0.0000 | 0.0000 | - | No | HCM brother. ICD implanted (syncope) |
| 33 | 61 | F | White | p.Gln543Lysfs*29  c.1626delG | 0.0000 | 0.0000 | - | No | Apical HCM and HF death brother |
| 34 | 25 | M | White | p.Gln543Lysfs*29  c.1626delG | 0.0000 | 0.0000 | - | No |  |
| 35 | 44 | M | White | p.Arg1059*  c.3175C>T | 3.94x10^-5^ | 7.35 x10^-5^ | Conflicting interpretations of pathogenicity | No |  |
| 36 | 73 | F | White | p.Glu1146Glyfs*12  c.3436-3437insG | 0.0000 | 0.0000 | - | No | HCM sister |
| 37 | 66 | M | White | p.Glu1179Argfs*93  c.3535delG | 6.58x10^-6^ | 1.47 x10^-5^ | - | No | HCM son |
| 38 | 73 | F | White | p.Lys184*  c.550A>T | 0.0000 | 0.0000 | - | No | HCM sister and nephew |
| 39 | 60 | M | White | p.Glu682*  c.2044G>T | 6.58x10^-6^ | 1.47 x10^-5^ | - | *MYH7* P (p.Arg869Cys) | HCM son |
| 40 | 67 | M | White | p.Trp1563*  c.4689delG | 0.0000 | 0.0000 | - | No | SCD son |
| 41 | 37 | F | White | p.Trp1563*  c.4689delG | 0.0000 | 0.0000 | - | No |  |
| 42 | 64 | M | White | p.Glu1098*  c.3292G>T | 0.0000 | 0.0000 | - | No |  |
| 43 | 42 | M | White | p.Glu1098*  c.3292G>T | 0.0000 | 0.0000 | - | No | HCM father |

Abbreviations: DCM: dilated cardiomyopathy; F: female; fs: frameshift; HF: heart failure; HCM: hypertrophic cardiomyopathy; ICD: implantable cardioverter defibrillator; LVH: left ventricular hypertrophy; M: male; P: pathogenic; SCD: sudden cardiac death; VUS: variant of unknown significance.

**Supplementary table 5.** Affected relatives (with a phenotype fulfilling HCM criteria).

| **Family** | **Age diagnosis** | **Sex (M: male, F: female)** | **ECG** | **Echocardiogram** | **Cardiac MRI** | **Creatine kinase** | **Holter – AF or NSVT** | **ICD** | **ASA/Myectomy** | **Transplant/**  **Mortality** |
| --- | --- | --- | --- | --- | --- | --- | --- | --- | --- | --- |
| 36 | 81 | F | SR, LVH, TWI V4-V6, DI, aVL, pathological Q wave DIII | ASH, reverse curvature, MLVWT 21mm, LVEF 70%, mid-cavity obstruction, no LVOTO | ASH, reverse curvature, MLVWT 21mm, LGE basal anteroseptum | 46 | NA | No | No | No |
| 37 | 42 | M | SR, IRBBB | ASH, sigmoid, MLVWT 13mm, LVEF 65%, no LVOTO | ASH, sigmoid, MLVWT 12mm |  | No | No | No | No |
| 32 | 40 | M | SR, left precordial TWI | ASH sigmoid, MLVWT 30mm; LVEF 65%, no LVOTO | NA |  | NA | Yes (syncope) | No | No |
| 32 | 76 | M | SR, LVH left precordial TWI | ASH sigmoid, MLVWT 19mm, LVEF 60%, no LVOTO | NA |  | NA | No | No | No |
| 32 | 46 | M | SR, LVH left precordial TWI | Concentric, MLVWT 13mm, LVEF 65%, no LVOTO | NA |  | NA | No | No | No |
| 18 | 59 | M | SR, LBBB | Concentric, MLVWT 17mm, LVEF 60% (2007), no LVOTO | Concentric, MLVWT 15mm, LVEF 22% (2019), extensive LGE intramiocardial and subepicardical anteroseptal, inferoseptal, basal-medium anterior e inferior, circunferencial subepicardial medio-apical | 154 | Yes, AF and NSVT | Yes | No | Admission for HF |
| 38 | 76 | F | SR | ASH, reverse curve, MLVWT 15mm, LVEF 66%, no LVOTO | ASH, reverse, MLVWT 15mm, LVEF 83%, no LGE | 42 | No | No | No | No |
| 23 | 62 | M | SR, LVH, TWI DI, aVL, V4-V6, pathological Q wave aVL | ASH, reverse curvature, MLVWT 24mm, LVEF 69%, LVOTO, RVH | NA |  | NA | No | No | No |
| 43 | 76 | M | SR, LVH, LAD, RBBB | Apical hypertrophy | NA | NA | No | No | No | No |

Abbreviations: AF: atrial fibrillation; ASH: asymmetrical septal hypertrophy; ASA: alcohol septal ablation; CRT-D: cardiac resynchronization therapy with defibrillator; ETT: exercise treadmill test; HF: heart failure; ICD: implantable cardioverter defibrillator; LA: left atria; LAHB: left anterior hemiblock; LBBB: left bundle branch block; LGE: late gadolinium enhancement; LV: left ventricle; LVEF: left ventricular ejection fraction; LVH: left ventricular hypertrophy; LVOTO: left ventricular outflow tract obstruction; MLVWT: maximal left ventricular wall thickness; MRI: Magnetic resonance imaging; NSVT: non-sustained ventricular tachycardia; NA: not available; RBBB: right bundle branch block; RV: right ventricle; RVH: right ventricular hypertrophy; SCD: sudden cardiac death; SR: sinus rhythm; TWI: T wave inversion.

**Supplementary figure 1.** Cardiac magnetic resonance imaging showing the prevalent phenotype of severe mid to apical hypertrophy and extensive late gadolinium enhancement (LGE) in 3 patients. Left to right: 4 chamber view end-diastole cine image, 4 chamber view LGE image, short axis LGE image.


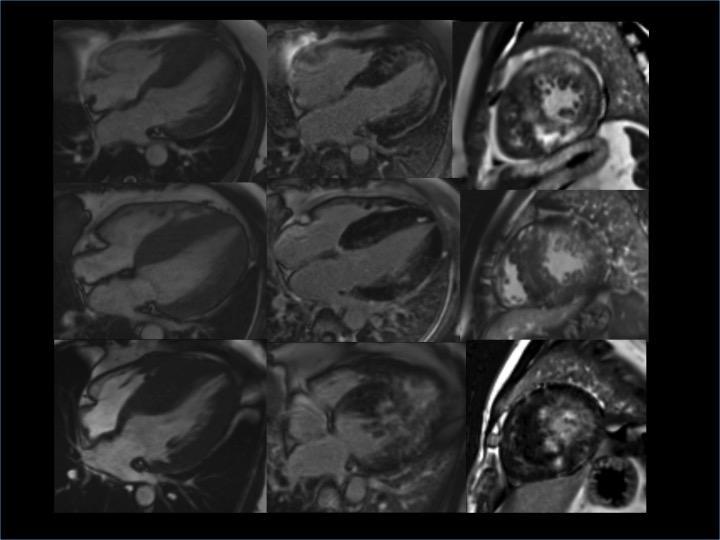


**Supplementary figure 2.** Immunostaining for plakoglobin (A) and desmin (B) in patient #23 showing normal distribution pattern and intensity of staining.


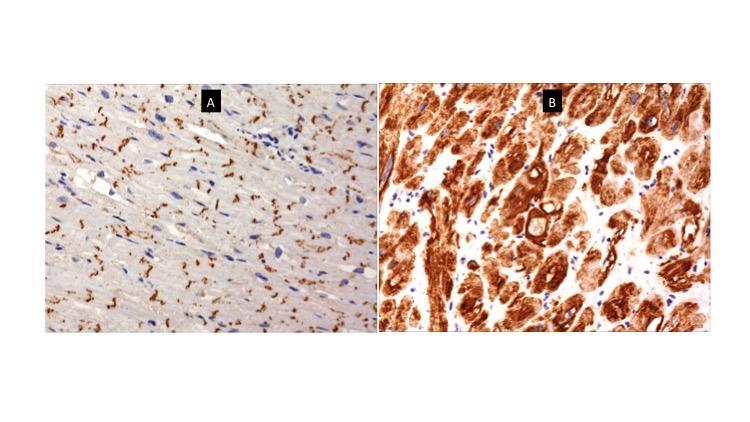


**Supplementary figure 3.** Histopathology of index patient #8. High power view of endomyocardial biopsy stained with Masson Trichrome. The myocytes are cut in their short axis and some show irregular clearing of the cytoplasm with displacement of the contractile elements to the periphery to give irregular small vacuoles. PAS staining was negative.


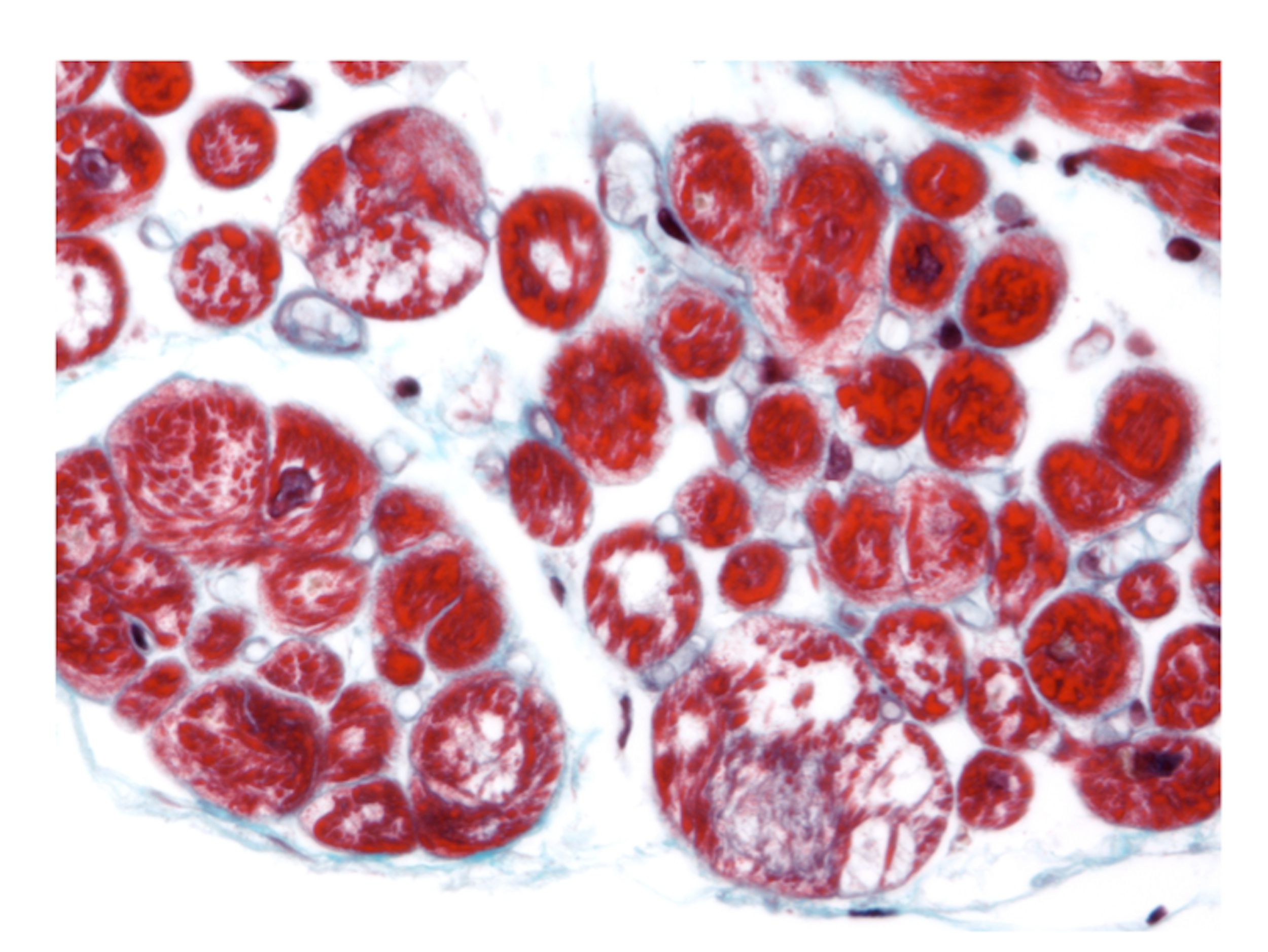


**SUPPLEMENTARY REFERENCES**

1. McKenna WJ, Spirito P, Desnos M, Dubourg O, Komajda M. Experience from clinical genetics in hypertrophic cardiomyopathy: proposal for new diagnostic criteria in adult members of affected families. Heart 1997;**77**(2):130-2.

2. Richards S, Aziz N, Bale S, Bick D, Das S, Gastier-Foster J, Grody WW, Hegde M, Lyon E, Spector E, Voelkerding K, Rehm HL, Committee ALQA. Standards and guidelines for the interpretation of sequence variants: a joint consensus recommendation of the American College of Medical Genetics and Genomics and the Association for Molecular Pathology. Genet Med 2015;**17**(5):405-24.

3. Surawicz B, Childers R, Deal BJ, Gettes LS, Bailey JJ, Gorgels A, Hancock EW, Josephson M, Kligfield P, Kors JA, Macfarlane P, Mason JW, Mirvis DM, Okin P, Pahlm O, Rautaharju PM, van Herpen G, Wagner GS, Wellens H, American Heart Association E, Arrhythmias Committee CoCC, American College of Cardiology F, Heart Rhythm S. AHA/ACCF/HRS recommendations for the standardization and interpretation of the electrocardiogram: part III: intraventricular conduction disturbances: a scientific statement from the American Heart Association Electrocardiography and Arrhythmias Committee, Council on Clinical Cardiology; the American College of Cardiology Foundation; and the Heart Rhythm Society: endorsed by the International Society for Computerized Electrocardiology. Circulation 2009;**119**(10):e235-40.

4. Monserrat L, Elliott PM, Gimeno JR, Sharma S, Penas-Lado M, McKenna WJ. Non-sustained ventricular tachycardia in hypertrophic cardiomyopathy: an independent marker of sudden death risk in young patients. J Am Coll Cardiol 2003;**42**(5):873-9.

5. Elliott PM, Poloniecki J, Dickie S, Sharma S, Monserrat L, Varnava A, Mahon NG, McKenna WJ. Sudden death in hypertrophic cardiomyopathy: identification of high risk patients. J Am Coll Cardiol 2000;**36**(7):2212-8.
